# Infusion of Induced Regulatory T Cells Alleviates Atherosclerosis by Reducing Pathological Macrophage‐Like Vascular Smooth Muscle Cells

**DOI:** 10.1002/mco2.70439

**Published:** 2025-10-22

**Authors:** Ximei Zhang, Ye Chen, Yesheng Ling, Lin Wu, Dinghui Liu, Linli Wang, Yong Liu, Guangyao Shi, Bin Zhou, Baoshun Hao, Zhenda Zheng, Shujie Yu, Min Wang, Jun Zhao, Donglan Zeng, Julie Wang, Yan Lu, Jun Tao, Wenhao Xia, Song Guo Zheng, Xiaoxian Qian

**Affiliations:** ^1^ Department of Cardiology Third Affiliated Hospital of Sun Yat‐Sen University Guangzhou China; ^2^ Department of Immunology School of Cell and Gene Therapy Songjiang Research Institute and Songjiang Hospital Affiliated to the Shanghai Jiaotong University School of Medicine Shanghai China; ^3^ Department of Clinical Immunology Third Affiliated Hospital of Sun Yat‐Sen University Guangzhou China; ^4^ Department of Hypertension and Vascular Disease First Affiliated Hospital of Sun Yat‐Sen University Guangzhou China

**Keywords:** atherosclerosis, iTregs, phenotypic switching, VSMCs

## Abstract

Atherosclerosis remains the primary driver of cardiovascular and cerebrovascular morbidity and mortality. A pivotal event in its pathogenesis is the phenotypic conversion of vascular smooth muscle cells (VSMCs), particularly the transition from a contractile to a macrophage‐like state. Using a murine model of atherosclerosis, we demonstrate that this process is orchestrated by a progressively disrupted perivascular immune milieu characterized by an expansion of CD44⁺ memory CD4⁺ T cells at the expense of CD44^−^ naive CD4⁺ T cells. Within this niche, CD44⁺ natural regulatory T cells (nTregs) actively promote VSMCs macrophage‐like reprogramming, whereas their CD44^−^ counterparts exert an opposing, protective effect. Reciprocally, macrophage‐like VSMCs foster the trans‐differentiation of nTregs into pathogenic Th17 cells, amplifying vascular inflammation. In contrast, induced Treg cells (iTregs) display phenotypic stability and potently inhibit VSMCs macrophage‐like switching, restrict pathological VSMCs migration, and curtail VSMCs survival. Systemic infusion of iTregs selectively remodels the perivascular immune microenvironment toward an antiatherogenic profile. Adoptive transfer of iTregs at early disease stages decreased the abundance of macrophage‐like VSMCs, attenuated plaque burden, and these benefits were partially mediated by transforming growth factor β signaling. Collectively, iTreg‐based cellular therapy represents a promising strategy to intercept VSMCs macrophage‐like transformation and limit atherosclerotic progression.

## Graphical abstract

Macrophage‐like phenotype switched VSMCs increase the trans‐differentiation of nature regulatory T cells (nTregs) to pathogenic Th17 cells. Induced regulatory T cells (iTregs) are stable and can inhibit the macrophage‐like phenotypic switching of VSMCs, as well as reduce pathological VSMC migration and survival. Adoptive transfer of iTregs to atherosclerotic mice reduced macrophage‐like phenotypic switching of VSMCs, inflammation and alleviated atherosclerosis, which was partly dependent on TGF‐β signaling.

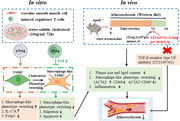

## 1 Introduction

Despite major therapeutic advances and primary prevention, atherosclerotic cardiovascular and cerebrovascular diseases remain the leading cause of death worldwide [1, 2, 3]. Chronic unresolved inflammation of the vascular wall is the pathophysiological basis for progressive atherogenesis and its complications [4, 5, 6]. Vascular smooth muscle cells (VMCs) are a major cell type in the atherosclerotic intima and play a crucial role in almost all stages of atherosclerotic plaque development [7, 8]. VSMCs invade the early atherosclerotic lesion from the media to expand lesions and form a protective fibrous cap to cover the necrotic core [8, 9]. Hence, VSMCs have been viewed as a plaque‐stabilizing parameter, and decreased VSMCs plaque content is associated with increased plaque vulnerability. However, the emergence of lineage‐tracing and transcriptomic studies has demonstrated that VSMCs comprise a much larger proportion of atherosclerotic plaques than originally thought [10]. Within the plaque, VSMCs shift from a contractile phenotype to a synthetic phenotype, leading to enhanced cell proliferation, migration, dedifferentiation, and inflammation [11]. In particular, VSMCs lose their own lineage markers and adopt alternative phenotypes, including macrophage‐like CD68, MAC‐2, MAC‐3, and foam cell‐like phenotypes [9, 10, 12].

Accumulating of cholesterol (Chol)‐enriched macrophage foam cells in the arterial wall is a hallmark of atherosclerotic plaque development [13, 14]. Macrophages in the atherosclerotic plaque are heterogeneous due to their diversity sources [15]. It is generally accepted that part of plaque macrophages originate from circulating monocytes [16]. Local proliferation of macrophage is also suggested to contribute to lesion macrophages, playing multifaceted roles in different stages of atherosclerosis [17]. Recent data suggest that trans‐differentiation of smooth muscle cell (SMC) is also an important contributor to plaque macrophages [9]. It was reported that substantial macrophages or foam cells in human or mouse atherosclerotic plaques are of VSMCs origin [7, 18, 19, 20]. These VSMCs undergo phenotype transition to macrophage‐like and foam cell‐like cells in atherosclerotic lesions, accumulating excess Chol, sustaining vascular wall inflammation and matrix remodeling, and eventually contributing to the development and progression of atherosclerosis [9, 12].

Recent single‐cell transcriptomic analyses have revealed that in atherosclerotic aortas, CD3e^+^ T‐cells constitute approximately 49% of the leukocyte population, CD19^+^ B‐cells account for 27%, and Itgam^+^ (CD11b^+^) myeloid cells represent 22% [21]. Similarly, in human atherosclerotic plaques, single‐cell RNA sequencing has identified T cells as comprising 52.4% of the leukocyte population [22]. Regulatory T cells (Tregs) are an important CD4^+^ T cell subpopulation that maintains immune tolerance and prevents autoimmune responses [23, 24]. Tregs are involved in atherosclerosis by suppressing inflammatory myeloid cells and educating macrophages to promote a tissue reparative environment [25, 26]. However, circulating and atherosclerotic plaque Tregs decline in the later stage of atherosclerosis, whereas total CD4^+^ effector T cells and splenic Tregs expand with increased atherosclerotic lesion size [27, 28]. Additionally, Tregs lose their capacity to protect against the progression of atherosclerosis when they were trans‐differentiated to Th1 or Th17 cells [29, 30, 31]. Indeed, endogenous Tregs generated in vivo are unstable in inflammatory environments [30, 32]. However, increased expression of Tregs artificially alleviated atherosclerosis, and depletion of CD4^+^ Tregs accelerated atherosclerosis [33, 34], implying that Tregs could still control the progression of atherosclerosis. The forkhead family transcription factor forkhead box protein P3 (Foxp3) is used as a specific molecular marker of Tregs. Foxp3‐positive Tregs are broadly classified into natural Tregs (nTregs) generated in vivo, which are derived from the thymus (tTreg) or extra‐thymically at peripheral sites (pTregs). Unlike nTreg, induced Tregs (iTregs) differentiated in vitro in cell culture in the presence of transforming growth factor β (TGF‐β) [24, 35]. Our previous study showed that iTregs are more stable under inflammatory conditions and can directly target inflammatory synovial fibroblasts to alleviate arthritis [36, 37, 38]. As phenotype switched VSMCs were reported to exhibit biological characteristics similar to those of synovial fibroblasts, including increased migration, proliferation, antiapoptosis, and inflammation, we wondered whether iTregs also affect pathological phenotypic switching, especially macrophage‐like switching of VSMCs. The answer to this question will greatly help determine the effect of Tregs on phenotype switched VSMCs during atherosclerosis.

In the current study, we aimed to investigate the complex interplay between VSMCs and the immune system in the context of atherosclerosis. Our findings revealed a novel mechanism by which VSMCs that have undergone macrophage‐like phenotypic switching play a critical role in the progression of atherosclerosis. Specifically, we demonstrated that these macrophage‐like phenotype switched VSMCs significantly increase the trans‐differentiation of Tregs to pathogenic Th17 cells, thereby exacerbating the inflammatory response and contributing to the development of atherosclerotic lesions. Furthermore, our research highlighted the potential of iTregs as a therapeutic intervention. We found that iTregs are not only stable but also capable of inhibiting the macrophage‐like phenotypic switching of VSMCs (macrophage‐like VSMCs), as well as reducing pathological VSMCs migration and survival. This therapeutic effect was partially dependent on TGF‐β signaling. Overall, our study provides new insights into the role of VSMCs in atherosclerosis and highlights the potential of iTregs adoptive transfer as a novel therapeutic strategy. This approach may emerge as a promising avenue for combating the macrophage‐like transformation of VSMCs, thereby offering a new direction for the treatment and management of atherosclerosis. Further research is warranted to explore the full potential of this therapeutic intervention and to better understand the underlying mechanisms involved.

## 2 Results

### 2.1 Macrophage‐Like VSMCs Reduce the Stability of nTregs but Not iTregs

nTregs are known to reduce atherosclerosis by licensing the proresolving function of macrophages [39] and maintaining immunological balance such as that between nTregs and Th17 cells [40]. However, nTregs lose their function in atherosclerosis, transforming into proatherogenic pathological T cells such as Th1 and Th17 cells [29, 31]. As macrophage‐like phenotypic switching of VSMCs comprises a major component of “macrophages” in the atherosclerotic environment, it is important to determine the interaction between nTregs and the phenotype switched VSMCs, especially macrophage‐like VSMCs. Since nTregs generated in vivo are reported to be unstable in atherosclerosis [28], we first investigated whether nTregs are able to maintain their phenotype when they meet macrophage‐like VSMCs.

Previous researches have demonstrated that Chol loading can directly induce the transformation of VSMCs into macrophage‐like cells, as evidenced by the upregulation of macrophage‐related genes such as *Cd68* and *Lgals3*, while downregulating smooth muscle‐specific genes like *Acat2* and *Myh11* [8, 41]. Moreover, recent transcriptome analysis has also revealed that Chol loading is the initial step in triggering a proinflammatory state in VSMCs [42]. Furthermore, while oxidized low‐density lipoprotein (ox‐LDL) primarily induces the trans‐differentiation of VSMCs into macrophage‐like cells through oxidative stress and inflammation [43], Chol can also drive phenotypic switching via lipid accumulation and subsequent alterations in cellular signaling pathways [8]. Therefore, in the current study, Chol was chosen instead of ox‐LDL to focus on the broader effects of lipid accumulation on the early stages of VSMCs phenotypic switching, without the confounding effects of oxidative stress that are often associated with ox‐LDL. Consistent with previous studies, we observed that hydrated Chol (Sigma; C4951) stimulation significantly prompted VSMCs to express the macrophage‐associated marker MAC‐2 and the formation of foam cells (Figure S1A). At the transcriptional level, we also noted that Chol stimulation facilitated the expression of macrophage marker‐related genes, including *Cd68*, *Lgals3*, and *Lgals2*, as well as inflammatory cytokine genes such as *Mcp1* and *Il6*; concurrently, it inhibited the expression of genes associated with the normal VSMC phenotype, including *Acta2*, *Calponin*, and *Tropoelastin* (Figure S1B).

**FIGURE 1:**
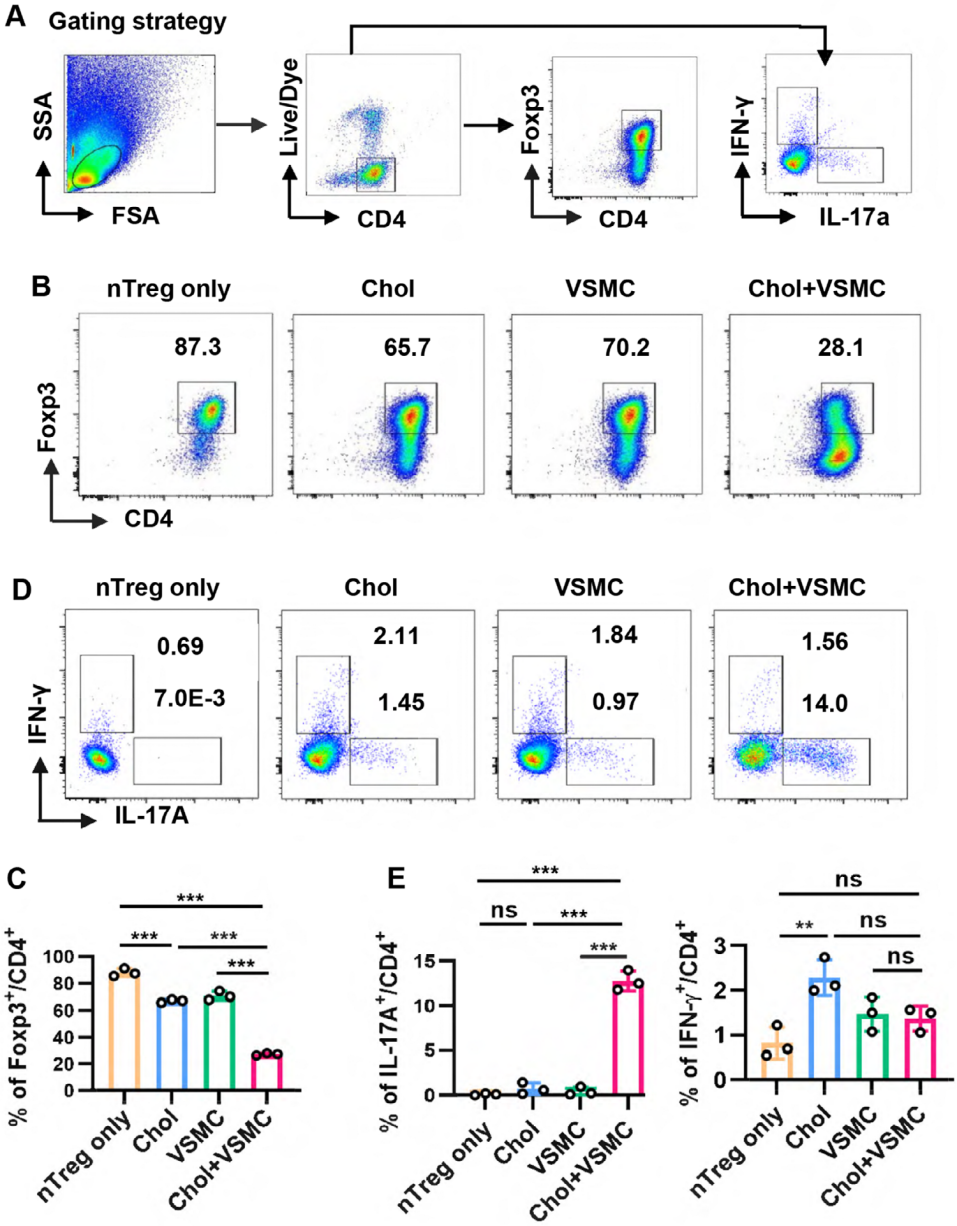
Macrophage‐like VSMCs reduce the stability of nTregs. After pretreated with water‐soluble Chol for 24 h, VSMCs were then cocultured with nTregs in the presence of Chol for another 72 h (Chol + VSMC). nTregs cultured alone (nTreg only) or in the presence of Chol (Chol), nTregs cocultured with VSMCs in the absence of Chol (VSMC) were used as controls. Nonadherent T cells were collected gently and stained with flow cytometric antibody of CD4, Foxp3, IFN‐γ, and IL‐17A. (A) Cells were gated on alive CD4^+^ T cells followed by analyzing the expression of Foxp3, IFN‐γ, and IL‐17A in CD4^+^ T cells as showed, the gating strategy employed during flow cytometric analysis is illustrated in panel A. (B and C) Expression of Foxp3 in CD4^+^ T cells from different groups was showed. (D and E) Expression of IFN‐γ and IL‐17A in CD4^+^ T cells from different groups was showed. Data are presented as the mean ± SD. Statistical significance was determined by one‐way ANOVA followed by Sidak's multiple comparison test. **p < 0.01, ***p < 0.001, ns = no significance. *n* = 3.

**FIGURE 2:**
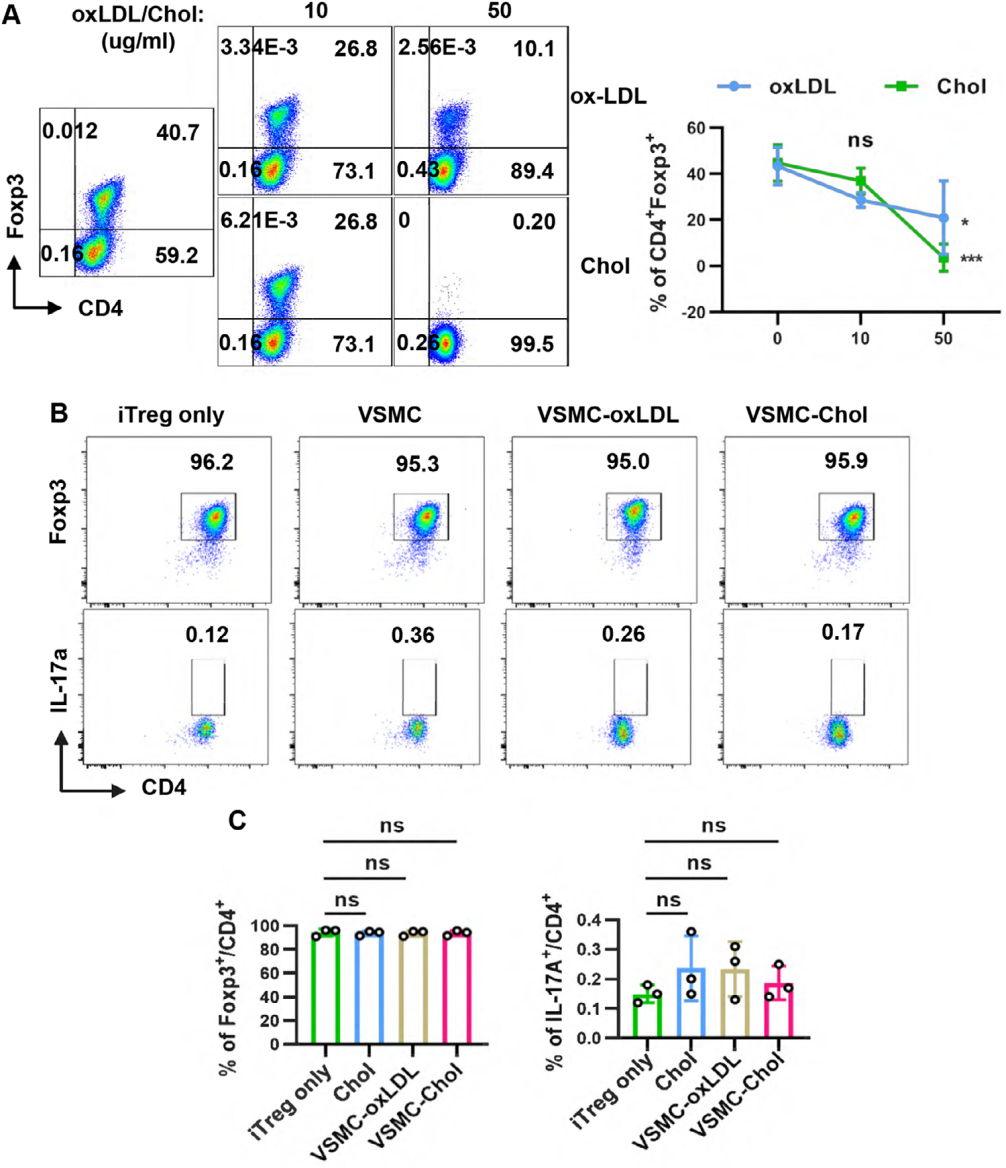
iTregs are stable in the presence of macrophage‐like VSMCs. (A) iTregs were induced from naive CD4^+^ T cells in the presence of Chol or ox‐LDL for 72 h, expression of Foxp3 in CD4^+^ T cells was analyzed by flow cytometry, 0 µg/mL versus 10 µg/mL and 0 µg/mL versus 50 µg/mL were compared. Statistical significance was determined by two‐way ANOVA followed by Sidak's multiple comparisons test. (B and C) Purified iTregs were cocultured with VSMCs in the presence of Chol or ox‐LDL for 72 h (VSMC‐Chol or VSMC‐oxLDL respectively), purified iTregs cultured alone (iTreg only) or cocultured with VSMCs (VSMC) were used as control. Expression of Foxp3 and IL‐17a in CD4^+^ T cells from different groups was analyzed by flow cytometry. Statistical significance was determined by one‐way ANOVA followed by Sidak's multiple comparisons test. Data are presented as the mean ± SD, *p < 0.05, ***p < 0.001, ns = no significance. *n* = 3.

We conducted coculture experiments to investigate the effect of macrophage‐like VSMCs on the stability of nTregs. Our findings revealed that a significant proportion of nTregs lost Foxp3 expression after coculture with VSMCs that had adopted a macrophage‐like phenotype, compared with nTregs cultured either alone regardless of Chol stimulation or cocultured with VSMCs without Chol stimulation (Figure 1A–C). Additionally, we observed an increased percentage of nTregs transitioning to Th17 cells following coculture with these macrophage‐like VSMCs (Figure 1A,D,E). Importantly, nTregs were found to significantly enhance the conversion of VSMCs to a macrophage‐like phenotype (Figure S2). However, while both Chol and ox‐LDL inhibited iTregs induction in vitro (Figure 2A), iTregs maintained their Foxp3 expression and did not trans‐differentiate into Th17 cells when cocultured with macrophage‐like VSMCs (Figure 2B,C).

### 2.2 iTregs Inhibit Macrophage‐Like VSMCs Phenotypic Switching

**FIGURE 3:**
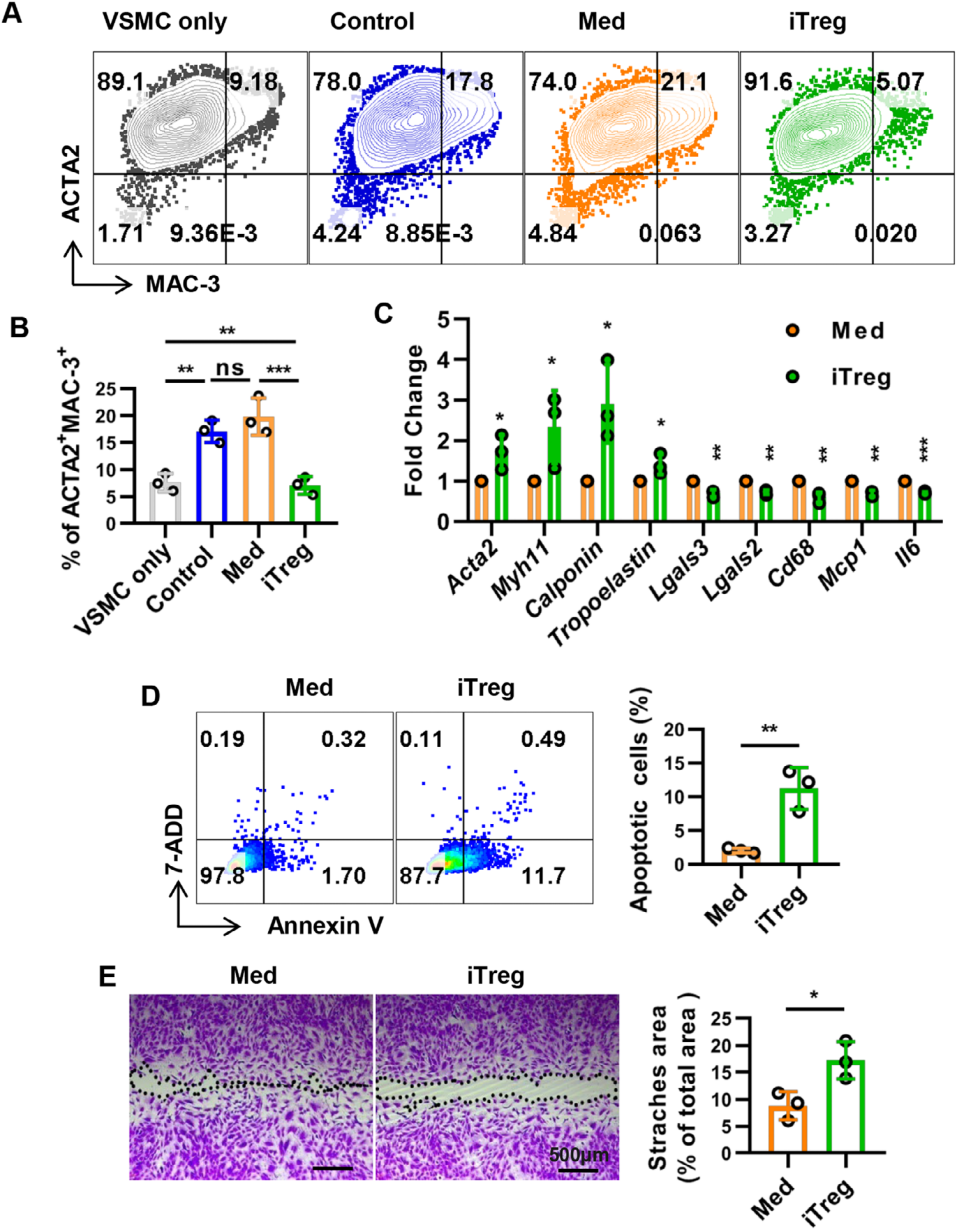
iTregs reduced macrophage‐like VSMCs phenotypic switching. VSMCs were cocultured with control cells (med) or iTregs in the presence ofChol for 48 h. VSMCs cultured alone with (control) or without the presence of Chol (VSMC only) were used as control. (A and B) VSMCs from different groups were collected and the protein expression of ACTA2 and MAC‐3 was analyzed by flow cytometry. (C) The gene expression of *Cd68*, *Lgals3*, *Lgals2*, *Mcp‐1*, *Il‐6*, *Acta2*, *Myh11*, *Calponin*, and *Tropoelastin* by VSMCs from different groups was detected by qPCR. The expression level of each gene was normalized to *Actb* or *Gapdh* and calculated as fold change relative to group of Med (Med = 1) using the 2^−ΔΔCt^ method. (D) VSMCs collected from different groups were subjected to Annexin V/PI labeling and analyzed by flow cytometry. (E) A scratch was made on confluence VSMCs, and then coculture with iTreg or Med in the presence of Chol for 24 h, the remaining scratch areas were analyzed after crystal violet staining. Data are presented as the mean ± SD. Statistical significance was determined by one‐way ANOVA followed by Sidak's multiple comparison test (four groups) or by Student's *t*‐test (two groups). *p < 0.05, **p < 0.01, ***p < 0.001, ns = no significance. *n* = 3.

To determine the crosstalk between iTregs and macrophage‐like VSMC trans‐differentiation, control cells (medium; Med) were applied as a vehicle control of iTregs to exclude nonspecific reactions when different types of cells were cultured together. We found that iTreg but not Med treatment significantly reduced macrophage‐like (expressing MAC‐3) phenotypic switching of VSMCs (Figure 3A,B). Moreover, in terms of mRNA levels, iTregs obviously reduced the expression of macrophage markers, including *Cd68*, *Lgals3*, and *Lgals2* by VSMCs, as well as inflammatory factor‐related genes such as *Mcp1* and *Il6*, while increasing the contractile phenotype‐related genes of VSMCs, including *Acta2*, *Myh11*, *Calponin*, and *Tropoelastin* (Figure 3C). These results highlight that iTregs reduce macrophage‐like phenotype conversion of VSMCs.

Since the atherogenic phenotype of VSMCs is invariably associated with increased cell viability and migration, we also examined the viability and migration of macrophage‐like VSMCs after coculture with iTregs. We also observed that a significantly higher proportion of VSMCs were found to undergo early apoptosis following coculture with iTregs in the presence of Chol, as compared with the control group (Figure 3D). Our results also support that VSMCs incubated with iTregs had markedly decreased migration ability (Figure 3E). In summary, these data indicate that iTregs can inhibit the migration and survival of macrophage‐like VSMCs.

### 2.3 Adoptive Transfer of iTregs Alleviates Atherosclerosis in ApoE^−/−^ Mice

To determine whether iTregs can reduce macrophage‐like VSMCs and alleviate atherosclerosis, iTregs were administered to ApoE**^−/−^** mice with atherosclerosis. We first observed the phenotype transformation of VSMCs at different time points during the course of atherosclerosis to determine the optimal timing for iTreg administration. Our data revealed that substantial VSMCs underwent phenotypic switching inside the plaque in the early stage of atherosclerosis (Figure S3). Interestingly, Foxp3‐positive nTregs gradually increased from 12 to 20 weeks of western diet (WD), implying an increased demand or pathological accumulation of nTregs as early as 12 weeks of WD (Figure S4). Thus, iTregs were adoptively transferred into mice after 12 weeks of WD.

We also noted that the balance of immune cells began to be disrupted after 12 weeks of WD, with more memory T cells (CD4^+^CD44^+^CD62L^−^) while less naive T cells (CD4^+^CD44^−^CD62L^+^), as well as more CD4^+^CD44^+^CD62L^−^ nTregs while less CD4^+^CD44^−^CD62L^+^ nTregs detected in the spleen and peri‐aortic lymph nodes (Figures S4 and S5). We further figure out whether CD44^+^CD62L^−^ nTregs and CD44^−^CD62L^+^ nTregs function differently on macrophage‐like VSMCs, especially on their ability to promote inflammation. To this end, CD44^+^CD62L^−^ nTregs and CD44^−^CD62L^+^ nTregs sorted respectively from Foxp3^GFP^ mice were cocultured with VSMCs under the condition for macrophage‐like phenotypic switching. Our data suggested that CD44^−^CD62L^+^ nTregs are superior to CD44^+^CD62L^−^ nTregs in inhibiting inflammatory and macrophage relative genes expression by VSMCs (Figure S6). In conclusion, we suppose that the atherosclerotic environment may increase the induction of nTregs, but most of these nTregs are not functional or even harmful, which maybe another reason why we need to bring in iTregs for atherosclerosis treatment.

**FIGURE 4:**
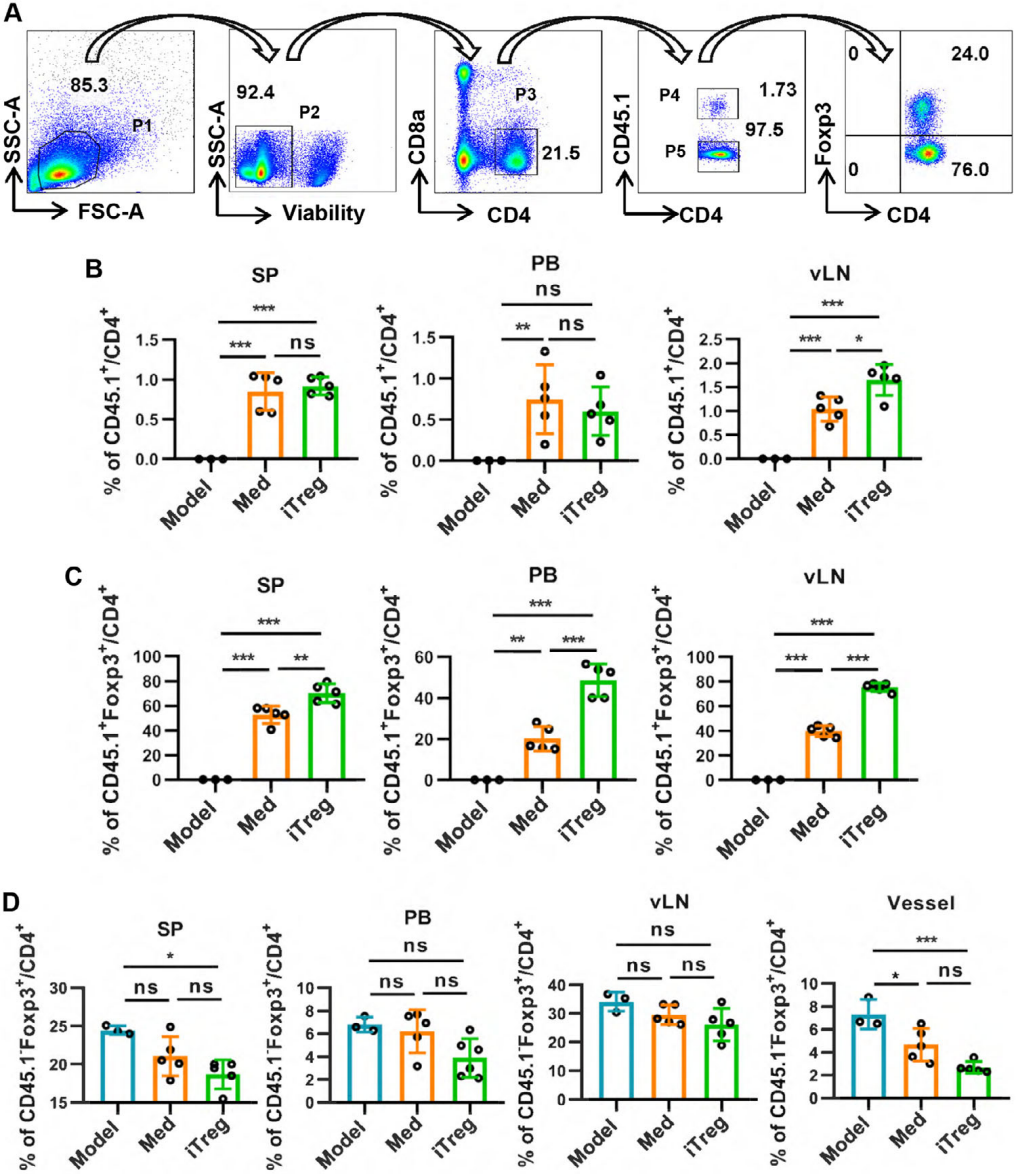
iTregs selectively migrate to peri‐aortic draining lymph nodes. ApoE^−/− ^mice were subjected to a western diet for 12 weeks and treated with CD45.1^+^ iTregs, followed by another 4 weeks of western diet before sacrificed. CD45.1, CD4, CD8a, and Foxp3 expressed by cells isolated from peri‐aortic draining lymph nodes (vLN), spleen (SP), peripheral blood (PB), and aortic tissue (vessel) was analyzed by flow cytometry. (A) The gating strategy employed during flow cytometric analysis is illustrated in panel A. (B) The expression of CD45.1^+^CD4^+^ cells as showed in P3 by cells from different tissues of different treated mice was showed. (C) The expression of CD45.1^+^Foxp3^+^ by CD4^+^ cells as showed in P4 by cells from different tissues of different treated mice was showed. (D) The expression of CD45.1^−^Foxp3^+^ by CD4^+^ cells as showed in P5 by cells from different tissues of different treated mice was showed. Data are presented as the mean ± SD. Statistical significance was determined by one‐way ANOVA followed by Sidak's multiple comparisons test. *p < 0.05, **p < 0.01, ***p < 0.001, ns = no significance. *n* = 3–6.

To further elucidate the role of iTregs in atherosclerosis, we initially assessed whether the injected iTregs could migrate to local vascular tissues. For this purpose, CD45.1^+^ mice were used as the source of iTregs or control cells. Our findings revealed that 3 weeks postinjection, a significantly higher percentage of adoptively transferred CD45.1^+^CD4^+^ T cells was detected in the peri‐aortic lymph nodes of mice treated with iTregs, compared with those treated with control cells (Figure 4A,B). Notably, no significant differences were observed in the spleens and peripheral blood between the two groups. Moreover, with iTreg treatment, a greater proportion of Foxp3‐positive cells, rather than Foxp3‐negative adoptive cells, migrated and settled in the peri‐aortic lymph nodes (approximately 50% before injection vs. approximately 75% in the peri‐aortic lymph nodes) (Figure 4A,C). Correspondingly, fewer CD45.1^+^Foxp3^−^ effector T cells were detected in mice treated with iTregs compared with those treated with control cells, particularly among those that migrated and settled in the peri‐aortic lymph nodes (Figure S7A,B). However, no significant differences were observed in the total CD45.1^−^ autologous T cells or CD45.1^−^ autologous effector T cells (Figure S7A,C,D). Interestingly, the expression of autologous CD45.1^−^ nTregs was reduced in the spleen and aortic tissues of mice treated with iTregs (Figure 4A,D). These results suggest that adoptively transferred iTregs selectively migrate to aortic tissues, where they may help restore immune balance, thereby reducing the frequency of nTregs. This selective migration and functional impact of iTregs provide valuable insights into their potential therapeutic role in atherosclerosis.

**FIGURE 5:**
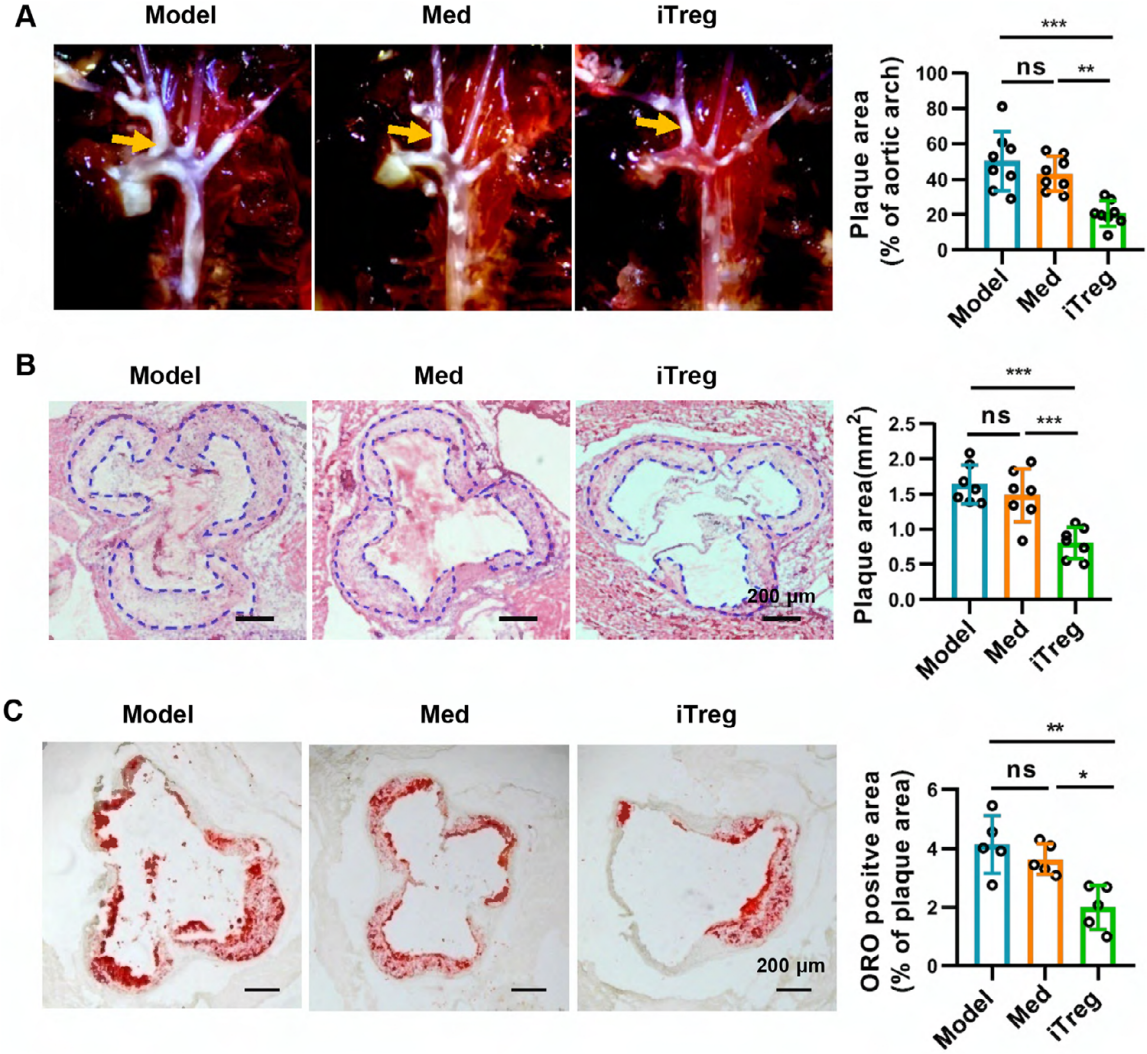
iTreg treatment alleviates atherosclerosis. ApoE^−/−^ mice were subjected to a western diet for 12 weeks and treated with iTregs/Med, followed by another 4 weeks of western diet before sacrifice. Shown are light microscopical images of aortic arch (A) or representative sections of aortic sinus stained with hematoxylin and eosin (HE) (B) or Oil Red O (ORO) (C). Data are presented as the mean ± SD. Statistical significance was determined by one‐way ANOVA followed by Sidak's multiple comparisons test. *p < 0.05, **p < 0.01, ***p < 0.001, ns = no significance. *n* = 5–8.

Compared with model mice (fed on WD diet receive no treatment) and mice that received control cells, mice treated with iTregs exhibited a significantly reduced lesion area ratio in the aortic arches and a smaller atherosclerotic lesion area in the aortic root (Figure 5A,B). Moreover, iTregs treatment also lower lipid contents in lesions (Figure 5C), although no significant changes in sera lipid contents were observed among the different groups (Figure S8). Furthermore, iTreg treatment also reduced the secretion of IL‐17a and IFN‐γ by draining lymph node cells (Figure S9). Taken together, these data indicate that iTregs inhibited the progression of atherosclerosis and reduced the systemic inflammatory responses in atherosclerotic mice.

### 2.4 iTregs Reduce Lesion Macrophage‐Like VSMCs in ApoE^−/−^ Mice

The pathogenesis of atherosclerosis involves the infiltration of macrophages from the lumen, the migration of VSMCs from the media into the intima, and the trans‐differentiation of VSMCs into macrophage‐like cells. Advanced macrophage‐like VSMCs may lose the expression of ACTA2, making it challenging to distinguish whether CD68⁺ACTA2^−^ cells are macrophages or VSMCs that have trans‐differentiated into a macrophage‐like phenotype. Therefore, in this study, only CD68⁺ACTA2⁺ cells are considered as macrophage‐like VSMCs. Given that previous reports have documented macrophages do not trans‐differentiate to express VSMCs markers, we hypothesize that cells within the plaque expressing both macrophage and VSMCs markers are VSMCs undergoing phenotypic switching to a macrophage‐like state.

Before assessing whether iTregs can reduce the formation of macrophage‐like VSMCs in plaques, we first evaluated whether CD4^+^ T cells could interact with VSMCs in vivo through direct contact. We used immunofluorescence to examine the spatial relationship between CD4^+^ T cells and VSMCs in the plaque. The results showed that VSMCs and CD4^+^ T cells are closely located in the fibrous cap region of the plaque, suggesting that Tregs may interact with VSMCs in this area (Figure S10A). We further assessed the migration of exogenous iTregs to the plaque using iTregs derived from Foxp3^GFP^ mice. Foxp3^GFP^ cells were detected in both the fibrous cap regions of the plaque and the adventitia, confirming that iTregs can indeed migrate and interact with VSMCs in the plaque (Figure S10B).

**FIGURE 6:**
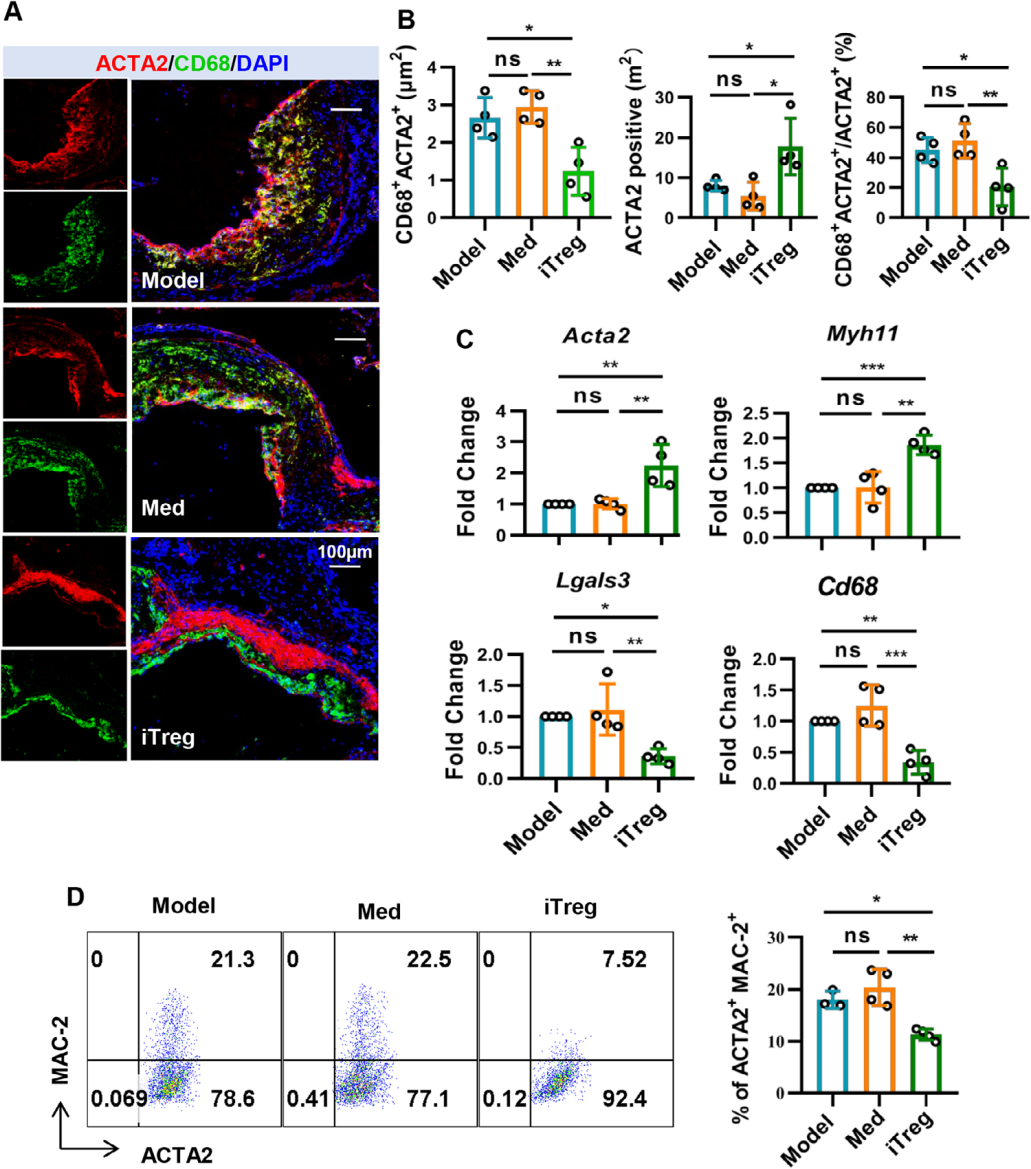
iTreg treatment reduce lesion macrophage‐like VSMCs in ApoE^−/−^ mice. ApoE^−/−^ mice were subjected to a western diet for 12 weeks and treated with iTregs/Med, followed by another 4 weeks of western diet before sacrifice. (A) Shown are representative immunofluorescence images of aortic sinus sections stained with 4′,6‐diamidino‐2‐phenylindole (DAPI), anti‐ACTA2, and anti‐CD68 antibodies. (B) The bar diagrams show the percentage of ACTA^+^, CD68^+^, and ACTA^+^CD68^+^ area as showed in the immunofluorescence images (*n* = 4). (C) Quantitative RT‐PCR analysis of the expression of macrophage‐like VSMCs related genes *Acta2*, *Myh11*, *Lgals3*, *Cd68* by aortic tissues (*n* = 4). The expression level of each gene was normalized to *Actb* or *Gapdh* and calculated as fold change relative to group of Model (Model = 1) using the 2^−ΔΔCt^ method. (D) Representative images of the expression ACTA2 and MAC‐2 by cells in the aortic tissues detected by flow cytometry. The bar diagram shows the statistical evaluation of the percentage of ACTA2 and MAC‐2 double positive cells in the aortic tissues (*n* = 3–4). Data are presented as the mean ± SD. Statistical significance was determined by one‐way ANOVA followed by Sidak's multiple comparisons test. *p < 0.05, **p < 0.01, ***p < 0.001, ns = no significance.

To examine the changes in cell components in atherosclerotic lesions, frozen sections from the aortic roots treated with iTregs or not were stained with CD68, MAC‐2, MAC‐3, and ACTA2 followed by immunofluorescence analysis. We found that iTreg treatment reduced the content of both CD68^+^ and CD68^+^ACTA2^+^ positive lesion “macrophages” while greatly increasing the population of ACTA2^+^ VSMCs (Figure 6A,B). Moreover, in terms of mRNA levels, iTregs treatment reduced the expression of *CD68*, *Lgals3* while increasing the expression of *Acta2*, *Myh11* (Figure 6C). We also observed a lower percentage of ACTA2^+^MAC‐2^+^ macrophage‐like VSMCs in the whole aorta tissues from mice treated with iTregs by flow cytometry analysis (Figure 6D). These findings suggest that iTreg treatment reduces macrophage‐like VSMCs in atherosclerotic plaques.

### 2.5 iTregs Reduce VSMC Phenotypic Switching Partly via TGF‐β Signaling

As indicated by our previous research and other studies, TGF‐β, a multifunctional cytokine, plays a crucial role in the development and immune regulatory function of Tregs [44, 45, 46]. Recent investigations have also highlighted the close association between TGF‐β and the phenotypic transformation of VSMCs [47]. It has been documented that VSMCs express TGF‐β receptors and that TGF‐β can facilitate the differentiation and maturation of VSMCs while preserving their contractile phenotype [48, 49, 50]. Furthermore, TGF‐β has the capacity to suppress the expression of KLF4, a critical factor in mediating this transformation process [47]. In light of these findings, we aimed to investigate whether the inhibitory effects of iTregs on the phenotypic switching of VSMCs are mediated through TGF‐β signaling. Consistent with our earlier studies, we found that over half of the Foxp3‐positive iTregs expressed the latency‐associated peptide (LAP), the inactive precursor of TGF‐β (Figure S11).

**FIGURE 7:**
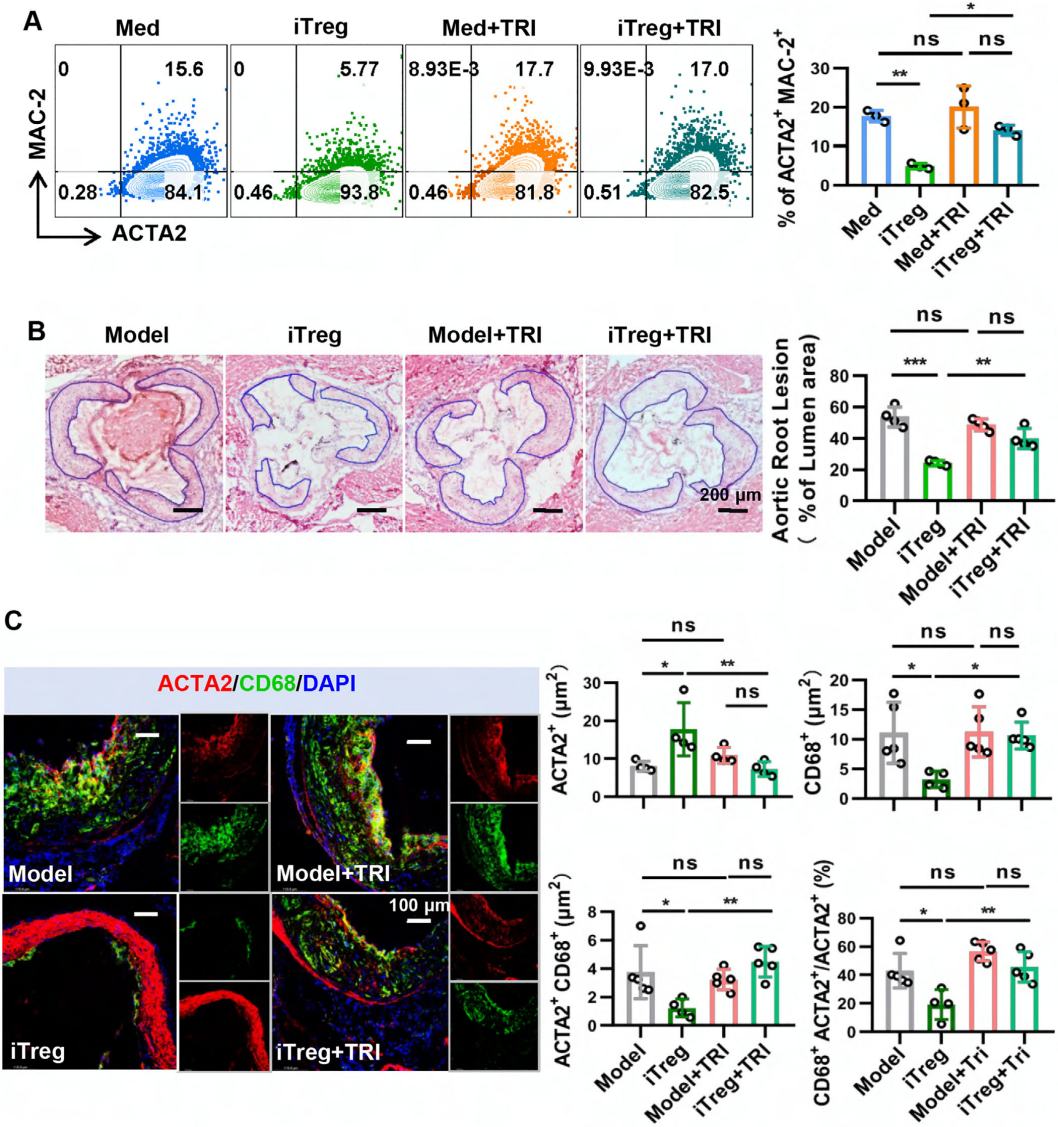
iTreg treatment reduces VSMCs phenotype switching partly via TGF‐β signaling. (A) VSMCs were cocultured with iTregs or control cells (Med) in the presence of Chol for 48 h, with or without blocking TGF‐β signaling by adding of TGF‐β receptor type I/II inhibitor (TRI, 2 µM, LY2109761), VSMCs from different groups were collected, and the protein expression of ACTA2 and MAC‐2 was analyzed by flow cytometry (*n* = 3). (B and C) ApoE^−/−^ mice were subjected to a western diet for 12 weeks followed by being treated with iTregs for another 4 weeks, with or without blocking TGF‐β signaling by intraperitoneal injection of TRI (LY2109761, 0.5 mg/mouse) every 3 days (starting immediately after iTreg injection and ending at the point of sacrifice). Representative images of aortic sinus stained with HE staining, the bar diagram shows the statistical evaluation of the plaque area (B) (*n* = 4). Representative immunofluorescence images of aortic sinus stained with DAPI, anti‐ACTA2, and anti‐CD68 antibodies, the bar diagram shows the percentage of ACTA^+^, CD68^+^ and ACTA^+^CD68^+^, ACTA^+^CD68^+^/ACTA2^+^ area as showed in the immunofluorescence images (C) (*n* = 4–5). Data are presented as the mean ± SD. Statistical significance was determined by one‐way ANOVA followed by Sidak's multiple comparisons test. *p < 0.05, **p < 0.01, ***p < 0.001, ns = no significance.

To further elucidate the involvement of secreted TGF‐β in the reduction of macrophage‐like VSMCs, we assessed the impact of various concentrations of TGF‐β on the trans‐differentiation of VSMCs. Our results revealed that higher levels of TGF‐β effectively diminished the expression of galectin‐3 (MAC‐2) in VSMCs and reduced the overall number of VSMCs (Figure S12). Given that iTregs continuously secrete TGF‐β and that this secretion may be modulated by cocultured VSMCs, we established a coculture system to further explore whether the inhibitory effect of iTregs on macrophage‐like VSMCs is dependent on TGF‐β signaling. Notably, the blockade of TGF‐β receptors using LY2109761 (2 µM) during coculture reversed the inhibitory effects of iTregs on the phenotypic switching of macrophage‐like VSMCs (Figure 7A). To further assess the reliance of iTreg‐mediated inhibition on TGF‐β signaling, we utilized an inhibitor of activin‐like kinase 5 (LY3200882, 5 µM) to specifically target the type 1 TGF‐β receptor and a neutralizing antibody against TGF‐β (MA5‐23795, 1 µg/mL). Our results indicated that while neither LY3200882 nor MA5‐23795 enhanced the phenotypic transition of VSMCs, both diminished the inhibitory effect of iTregs on the phenotypic transition of macrophage‐like VSMCs (Figure S13). Furthermore, LY3200882 was found to diminish the capacity of iTregs to induce apoptosis in VSMCs under conditions favoring phenotypic transition (Figure S14). Correspondingly, with TGF‐β receptors blocked, the therapeutic effect of iTregs on atherosclerosis and the phenotypic switching of VSMCs was also diminished significantly (Figure 7B,C). In summary, these findings substantiate that iTregs inhibit the formation of macrophage‐like VSMCs and attenuate atherosclerosis, at least in part through TGF‐β signaling pathways.

## 3 Discussion

VSMCs‐derived macrophage‐like cells induce a chronic inflammatory response within the plaque and constitute the main component of atherosclerosis [51]. The dual role of iTregs in modulating inflammation and the interactions between VSMCs and immune cells prompted us to investigate the contribution of iTregs to the macrophage‐like phenotype conversion of VSMCs in atherosclerosis [52, 53, 54]. Our results demonstrate that adoptive transfer of iTregs alleviates plaque progression by reducing macrophage‐like VSMCs phenotypic switching, at least partly via TGF‐β signaling. Moreover, iTregs reduce migration and promote apoptosis of these macrophage‐like VSMCs, which may also contribute to the alleviation of atherosclerosis.

Current research indicates that the migration of VSMCs into atherosclerotic plaques can have both beneficial and detrimental effects on plaque stability, depending on the stage of the disease. Initially, the migration of VSMCs may temporarily contribute to lipid uptake and reduce local inflammation by easing the lipid clearance burden on macrophages [55, 56]. Continuous inflammatory stimulation and lipid phagocytosis can trigger phenotypic switching in VSMCs, particularly a macrophage‐like phenotypic switch [57, 58]. This switch endows VSMCs with enhanced capabilities for cell proliferation, migration, and secretion of various inflammatory chemokines and cytokines, thereby accelerating the progression of atherosclerosis [57, 58]. Therefore, when exploring the inhibition of atherosclerotic plaque formation by interfering with the phenotypic switching of VSMCs, it is crucial to consider the stage of disease development.

In the study, we observed that macrophage‐like VSMCs reduce the stability of nTregs while iTregs remain stable in their presence. According to our study, this differential effect may be partly attributed to the different expression of receptor for IL‐6 between these two Treg subsets [36, 37, 59, 60, 61]. Macrophage‐like VSMCs can express inflammatory cytokines such as IL‐6, IL‐1β, and TNFα [9]. These cytokines further promote the phenotypic transformation of VSMCs [62, 63] and also influence the stability of nTregs [38]. Our previous research has shown that the expression of receptors for IL‐6, IL‐1β, and TNFα on nTreg and iTreg is different [38]_._ iTregs are more stable than nTregs, and this stability is closely related to the lower expression of IL‐6 receptors on iTregs compared with nTregs [38, 59]. Neutralizing IL‐6 or inhibiting the expression of IL‐6 receptor can enhance the stability of nTregs and inhibit their trans‐differentiation into Th17 cells via IL‐6/IL6R signaling [38]. In this study, we observed that the expression of IL‐6 was increased in macrophage‐like VSMCs, and iTregs were able to inhibit this expression, suggesting that IL‐6 is a key factor in macrophage‐like phenotype switching of VSMCs and IL‐6 secreted by macrophage‐like VSMCs may affect the stability of nTreg via IL‐6/IL6R signaling. We are committed to further exploring these mechanisms in our future studies.

Our research has demonstrated that iTregs possess the capacity to inhibit the migration of macrophage‐like VSMCs and to promote their apoptosis. Consequently, the adoptive transfer of iTregs at the early stages of atherosclerosis is emerging as both a crucial and appropriate preventive and therapeutic strategy, particularly prior to the initiation of VSMC migration and proliferation. In this study, we observed that iTregs selectively migrate to the peri‐aortic draining lymph nodes, which may significantly influence atherosclerosis progression through several mechanisms. First, iTregs are recognized for their pivotal role in maintaining immune homeostasis. Their migration to the peri‐aortic draining lymph nodes may directly modulate the activation and proliferation of effector T cell subsets, as well as other immune cells that contribute to plaque inflammation. This is particularly noteworthy, as we detected a reduction in the expression of nTregs in the aortic tissues of mice administered with iTregs, suggesting a potential restoration of immune balance. Second, iTregs are capable of producing anti‐inflammatory cytokines such as TGF‐β and IL‐10, while concurrently inhibiting the release of proinflammatory cytokines such as IL‐17a and IFN‐γ. This action may lead to a decrease in systemic inflammation and a reduction in the recruitment of inflammatory cells to atherosclerotic plaques, ultimately mitigating disease progression. Furthermore, the migration of iTregs to the perivascular lymph nodes enables them to regulate the local vascular microenvironment by secreting TGF‐β and other factors. This regulatory function may inhibit the migration of VSMCs toward the vascular intima and promote apoptosis in VSMCs that have undergone phenotypic transformation. We remain committed to further investigating these mechanisms in our future studies.

iTregs may exert their inhibitory effects on plaque formation through direct interference with the phenotypic transformation of VSMCs. Moreover, by modulating the immune balance and mitigating inflammation, iTregs may disrupt the release of chemotactic signals that induce VSMCs migration, thereby breaking the vicious cycle between inflammation and VSMCs phenotypic switching.

Indeed, our research group and others have previously demonstrated that iTregs generated ex vivo exhibit superior stability and functionality in inflammatory and high‐salt diet environments compared with nTregs generated in vivo [38, 64, 65, 66]. The stability of nTregs in the context of atherosclerosis has been the subject of limited investigation, with conflicting findings reported in the literature [67, 68]. However, certain studies have noted that Foxp3^+^RORγt^+^ nTregs in the gastrointestinal tract maintain stability and play a role in modulating aberrant Th2 responses [69]. Furthermore, given the distinct plasticity of Th17 cells across various inflammatory contexts [70], the role of IL‐17A in atherosclerosis is not unequivocal. Rather than being uniformly proatherosclerotic, IL‐17A has been suggested to either exert athero‐protective effects [71] or to have no impact on the disease process [72]. Consequently, elucidating the precise role of the trans‐differentiation of nTregs into IL‐17A‐expressing cells, as stimulated by macrophage‐like VSMCs, will be of considerable interest for future research endeavors.

Our findings revealed a progressive accumulation of memory CD4^+^ T cells in parallel with a corresponding decrease of naive CD4^+^ T cells during the early and middle stages of atherosclerosis progression. Concurrently, the expression Foxp3‐positive nTregs were observed to incrementally rise throughout the atherosclerotic process in mice. Administration of exogenously derived, relatively stable iTregs to these mice resulted in diminished plaque formation, reduced IL‐17A expression, and lowered endogenous Foxp3 expression. In conjunction with our in vitro research, which indicates that the phenotypic switching of VSMCs facilitates the differentiation of nTregs into Th17 cells, we postulate that alterations in T cell subsets within the immune cell repertoire may be instrumental in the pathogenesis of atherosclerosis. The pathological phenotypic transformation of VSMCs is posited to promote the trans‐differentiation of nTregs into pathogenic Th17 cells, which in turn may exacerbate the pathological phenotypic transformation of VSMCs. Exogenous supplementation with iTregs may ameliorate atherosclerosis by rectifying the interplay between VSMCs and pathogenic nTregs, thereby reducing Th17 cell differentiation and restoring the balance of T cell subsets. Further investigations into the interrelationship between the equilibrium of memory/naive T cells, nTregs/Th17 cells, and the occurrence of pathological macrophage‐like VSMCs phenotypic switching may prove beneficial in elucidating the underlying mechanisms of atherosclerosis.

Evidence indicates that TGF‐β is essential for the differentiation and functional maintenance of iTregs both in vitro and in vivo [73, 74]. Additionally, research has further demonstrated that TGF‐β is crucial for maintaining immune homeostasis and preventing autoimmune responses [74]. Consequently, the generation of global TGF‐β knockout (KO) mice raises concerns regarding their overall viability and health, thereby limiting the utility of TGF‐β KO iTregs in investigating the role of TGF‐β signaling in the phenotypic switching of VSMCs. To address this limitation, we utilized pharmacological inhibition of TGF‐β signaling in wild‐type iTregs by employing LY2109761, a dual inhibitor of TGF‐β receptor types I and II. Additionally, we applied LY3200882, which selectively inhibits TGF‐β receptor type I, along with a TGF‐β neutralizing antibody, to elucidate the molecular mechanisms through which iTregs govern phenotypic switching in VSMCs. Our findings revealed that both receptor blockade and direct neutralization of TGF‐β effectively reverse the inhibitory effects of iTregs on the phenotypic switching of VSMCs. In future studies, we plan to implement conditional KO strategies that will specifically ablate TGF‐β expression in T cells, rather than in a global manner across the entire organism. This approach may mitigate the potential impacts on overall mouse survival while still enabling us to investigate the role of TGF‐β signaling in the iTreg‐mediated phenotypic modulation of VSMCs.

Further experiments are needed to verify the exact role of macrophage‐like VSMCs phenotypic switching in atherosclerosis. In the current study, we demonstrated that a large number of cells weakly expressed both VSMCs markers and macrophage‐related markers in the plaque. Combined with the existing research, these results support that VSMCs undergoing macrophage‐like trans‐differentiation [75] express both VSMCs and macrophage markers in part. We employed these cells as representative of macrophage‐like VSMCs, which may underestimate the role of VSMCs in atherosclerosis and the function of iTregs in inhibiting macrophage‐like VSMCs phenotypic switching. Moreover, while some researchers hypothesize that macrophages do not differentiate into VSMCs lineages [76, 77], others consider that VSMCs marker‐positive cells within lesions are of myeloid origin [78, 79]. Further studies including lineage tracing are needed to further understand the role of macrophage‐like cells in atherosclerosis and whether this process is reversible. Therefore, the underlying mechanism(s) of macrophage‐like phenotype transformation of VSMCs also needs to be further elucidated.

In conclusion, this study elucidates the critical interplay between VSMCs and immune regulation in atherosclerosis progression. We demonstrate that macrophage‐like phenotypic switching of VSMCs not only promotes inflammatory responses but also facilitates the trans‐differentiation of nTregs into pathogenic Th17 cells, thus amplifying vascular inflammation. iTregs, characterized by their superior stability and functionality, effectively inhibit this macrophage‐like phenotypic conversion of VSMCs, reduce VSMCs migration, promote apoptosis of macrophage‐like VSMCs, and consequently attenuate plaque development. Importantly, these protective effects of iTregs are at least partially dependent on TGF‐β signaling. Our findings highlight the therapeutic potential of adoptive iTreg transfer as a promising strategy to restore immune homeostasis, inhibit detrimental VSMCs phenotypic switching, and ultimately mitigate the progression of atherosclerosis. Further exploration of the molecular mechanisms governing VSMCs plasticity and Treg stability will be essential for optimizing iTreg‐based interventions in cardiovascular diseases.

## 4 Materials and Methods

### 4.1 Mice

Mice were purchased from Beijing Vital River Laboratory Animal Technology Co., Ltd (Beijing, China). The mice were bred and maintained at the Guangdong Provincial Laboratory Animal Monitoring Institute under specific pathogen‐free and standard laboratory conditions. All animal procedures were performed in compliance with the procedures approved by the Animal Welfare Ethics Committee of the Guangdong Provincial Department of Science and Technology. To exclude possible hormonal effects (e.g., estrogen), only male mice were used in the experiments.

### 4.2 Atherosclerosis and Histopathology

Eight‐week‐old male apolipoprotein E knock out (ApoE^−/−^) mice were randomly distributed to each group and fed a WD with 1.25% Chol and 40% (w/w) fat (D12108C; Research Diets) for 15 weeks to induce atherosclerosis. For iTreg treatment, iTregs were induced as previously reported [80, 81]. Briefly, 2.0 × 10^6^ iTregs suspended in 200 µL sterile phosphate‐buffered saline (PBS) were injected intravenously after 12 weeks of WD, followed by another 3 weeks of WD before sacrifice. The mice treated with PBS only or control cells were used as the control or vehicle control, respectively. To block TGF‐β signaling, TGF‐β receptors type I/II inhibitor (LY2109761, 0.5 mg/mouse) was administered intraperitoneally every 3 days, starting immediately after iTreg injection and ending at the point of sacrifice. After 15 weeks of WD, the mice were sacrificed and the tissue samples were collected for the followed processing.

### 4.3 Characterization of Aortic Leukocytes and VSMCs

The whole aorta, from the ascending aorta to the bifurcation of the common iliac artery, was opened longitudinally and minced into 1–2 mm pieces in RPMI culture medium supplemented with 2% FBS. To characterize aortic leukocytes in aortic tissues, aortic pieces were resuspended in a fresh enzyme cocktail with collagenase type I (Sigma–Aldrich; C0130, 675 U/mL), collagenase type XI (Sigma–Aldrich; C7657, 187.5 U/mL), and hyaluronidase type I‐s (Sigma–Aldrich; H1115000, 9 U/mL) and incubated in a shaker at 37°C for 45 min. Following incubation, the samples were filtered through a 70‐µm strainer and centrifuged at 300×*g* for 5 min. The cell pellets were washed once with FACS buffer (2% FBS in PBS), stained with Fixable Viability Dye eFluor 780 (eBioscience; #65‐0865‐14) as well as fluorochrome‐conjugated antibodies and analyzed by flow cytometry [82]. For characterization of aortic VSMCs, aortic pieces were resuspended in an enzymatic solution containing 2 mg/mL of collagenase II (Sigma; C6885) in DMEM culture medium supplemented with 2% FBS and incubated in a shaker at 37°C for 30–45 min. Following incubation, the samples were filtered through a 70 µm strainer and centrifuged at 300×*g* for 5 min. The cell pellets were washed once with FACS buffer (2% FBS in PBS), stained with fluorochrome‐conjugated antibodies specific for mouse ACTA2, CD68, MAC‐2, and MAC‐3, and analyzed by flow cytometry [83].

### 4.4 Isolation and Culture of Primary VSMCs

Primary murine VSMCs were isolated from mouse aortas and were routinely maintained in DMEM supplemented with 10% serum as described in a previous study [83]. Briefly, aortas were obtained from 6 to 8 weeks old C57BL6/J mice. For adventitia removal, the aortas were incubated for 5 min in enzymatic solutions containing 2 mg/mL of collagenase II (Sigma; C6885) in DMEM culture medium supplemented with 2% FBS. The adventitia was then removed. Furthermore, the aortas were cut open longitudinally and the endothelium was removed by gentle scraping. The medial layer was minced into small pieces and fully digested into single cells by incubating via the same enzymatic method used for adventitia removal for 30 min. After filtering with a Cell Strainer (70 µm), the single‐cell were collected and resuspended culture in a complete DMEM culture medium (with 10% FBS, 1% penicillin/streptomycin) and incubated in a 5% CO_2_ atmosphere at 37°C. VSMCs were analyzed by flow cytometry following staining for a SMC marker using a mouse monoclonal antiactin, α‐smooth muscle‐FITC antibody (Sigma–Aldrich; F3777), to ensure that the purity of VSMCs was above 95%. VSMCs at passages 3–5 were used for experiments.

### 4.5 Naive CD4^+^ T Cell and iTreg

The peripheral lymph nodes and spleens were collected from C57BL/6 mice and ground to obtain single cells, before eliminating splenic erythrocytes using red blood cell lysis buffer (Sigma–Aldrich). Total T cells were enriched with nylon wool, followed by magnetic cell sorting with an auto magnetic cell sorter (MACS) (MiltenyiBiotec, Germany) to obtain purified naive CD4^+^ T cells. In brief, enriched T cells labeled with biotin anti‐CD8, CD25, B220, CD11b, CD11C, and CD49b antibodies and antibiotin microbeads were subjected to depletion, followed by positive selection with CD62L microbeads by auto MACS. The purity of naive CD4^+^ T cells was determined as CD4^+^CD25^+^CD62L^+^ using flow cytometry, and naive CD4^+^ T cells with purity >95% were used to induce iTregs or control cells (Med).

For iTreg induction, purified naive CD4^+^ T cells were cultured in the presence of anti‐CD3/CD28‐coated beads (cells to beads at a 1:5 ratio), rhIL‐2 (50 U/mL), and rhTGF‐β (2 ng/mL) in a 48‐well plate for 3 days to induce iTregs as previously described [84]. Cells cultured under a similar condition except the presence of rhTGF‐β served as control cells (Med). The percentage of Foxp3 expression was detected by flow cytometry and was found to be approximately 50% for iTregs and 10% for control cells.

For naive CD4^+^ T cells isolated from Foxp3^GFP^ C57BL/6 mice, iTregs were collected and labeled with fluorochrome‐conjugated antibodies specific for mouse CD4 antibodies, CD4^+^GFP^+^ cells were sorted by flow cytometry and used as purified iTregs. The purity of purified iTreg was determined as the expression percentage of CD4^+^Foxp3^+^ by flow cytometry and was approximately 95%.

### 4.6 nTreg Isolation

For isolation of nTregs, enriched T cells obtained from Foxp3^GFP^ C57BL/6 mice as showed above were labeled with fluorochrome‐conjugated antibodies specific for mouse CD4 antibodies and CD4^+^Foxp3^+^ T cells were sorted by flow cytometry and used as nTregs. The purity of the nTregs was determined as the expression percentage of CD4^+^ Foxp3^+^ by flow cytometry and was approximately 95%.

### 4.7 Treg Coculture with Macrophage‐Like VSMCs

VSMCs were treated with water‐soluble Chol (catalog no. C4951, 10 µg/mL) for 72 h to induce macrophage‐like VSMC phenotypic switching, as previously reported [85]. For coculture, VSMCs were pretreated with water‐soluble Chol for 24 h, and then cocultured with Tregs (Tregs:VSMCs = 10:1) or control cells with or without the presence of water‐soluble Chol. After 72 h of coculture, T cells and treated VSMCs were collected separately and labeled with different target proteins, followed by analyzing with flow cytometry.

### 4.8 Statistical Analysis

The data are presented as means ± standard deviation (SD) and were analyzed utilizing GraphPad Prism (GraphPad Software, San Diego, CA, USA). Statistical analyses and comparisons were conducted using Student's *t*‐test, one‐way analysis of variance (ANOVA), and for comparisons between two groups under different conditions, two‐way ANOVA was employed. A *p* value of less than 0.05 was deemed statistically significant.

Primers used in the study are listed in Table S1.

Detailed materials and methods are provided in the Supplementary Materials.

## Author Contributions

Ximei Zhang, Xiaoxian Qian, and Song Guo Zheng designed this study and protocol development. Ximei Zhang and Ye Chen were responsible for conducting the experiments. Ximei Zhang, Ye Chen, Yesheng Ling, Lin Wu, Dinghui Liu, Linli Wang, Jun Zhao, and Donglan Zeng were responsible for data analysis. Ximei Zhang conducted the manuscript writing. Song Guo Zheng, Xiaoxian Qian, Yong Liu, Guangyao Shi, Bin Zhou, Baoshun Hao, Zhenda Zheng, Shujie Yu, Min Wang, Julie Wang, Yan Lu, Jun Tao, and Wenhao Xia critically revised the manuscript. All the authors have read and approved the final manuscript.

## Conflicts of Interest

Author Song Guo Zheng is an Editorial board member of *MedComm*. Author Song Guo Zheng was not involved in the journal's review of or decisions related to this manuscript. The other authors declared no conflict of interest.

## Ethics Statement

The Animal Welfare Ethics Committee of the Guangdong Provincial Department of Science and Technology approved the animal procedures (approval no. SYXK(Yue)2016‐0122;2020.03.25‐2021.11.20), which were conducted following the ethical principles outlined in the NIH Guide for the Care and Use of Laboratory Animals.

## Supporting information




**Figure S1** Chol stimulation promote a macrophage‐like phenotype switching of VSMCs. VSMCs were stimulated with Chol (50 µg/mL) for 48 h to induce the transformation into a macrophage‐like phenotype. (A) The expression of macrophage‐like VSMCs was assessed by immunofluorescence staining of ACTA2 (red), MAC‐2 (green), DAPI (blue), and Oil Red O staining of foam cell formation. (B) The expression levels of VSMCs‐related marker genes *Acta2*, *Calponin*, and *Tropoelastin*, as well as macrophage‐related marker genes *Lagls2*, *Lagls3*, *Cd68* and inflammatory cytokine genes *Mcp1* and *Il6* were measured by quantitative PCR (qPCR). The expression level of each gene was normalized to *Gapdh* or *Actb* and calculated as fold change relative to group of VSMCs only (VSMC only = 1) using the 2^‐ΔΔCt^ method. Data are presented as mean ± SD. Statistical significance was determined by Student's *t*‐test. *p < 0.05, **p < 0.01, ns = no significance. *n* = 3.
**Figure S2** nTregs promote the phenotypic conversion of VSMCs. VSMCs were cocultured with nTregs in the presence of Chol for 48 h (Chol + VSMC + nTreg), VSMCs cocultured with nTregs with no Chol (VSMC + nTreg) or VSMCs cultured alone without (VSMC only) or with (VSMC + Chol) Chol stimulation were used controls. Expression of ACTA2 and MAC‐3 by VSMCs from different groups was analyzed by flow cytometry. Data are presented as the mean ± SD. Statistical significance was determined by one‐way ANOVA followed by Sidak's multiple comparisons test. *p < 0.05, **p < 0.01, ***p < 0.001, ns = no significance. *n* = 3.
**Figure S3** Phenotypic conversion of VSMCs in atherosclerosis. ApoE^−/−^ mice were subjected to a western diet for 12 weeks to 20 weeks, expression of ACTA2 (red), CD68 (green), and DAPI (blue) in the aortic root was detected by immunofluorescence staining.
**Figure S4** The balance of immune cells in atherosclerosis was disrupted. ApoE^−/−^ mice were subjected to a normal diet (ND) for 5 months or a western diet for 3–5 months, the expression of CD44, CD62L, CD4, and Foxp3 in cells isolated from spleens were detected by flow cytometry. (A) The gating strategy employed during flow cytometric analysis is illustrated in panel A. (B) Data in the bar graphs are presented as the mean ± SD. Statistical significance was determined by one‐way ANOVA followed by Sidak's multiple comparisons test. *p < 0.05, ***p < 0.001, ns = no significance. *n* = 3–4.
**Figure S5** The balance of immune cells in atherosclerosis was disrupted. ApoE^−/−^ mice were subjected to a normal diet (ND) for 5 months or a western diet for 3–5 months, the expression of CD44, CD62L, CD4, and Foxp3 in cells isolated from peri‐aortic lymph nodes were detected by flow cytometry. (A) The gating strategy employed during flow‐cytometric analysis is illustrated in panel A. (B) Data in the bar graphs are presented as the mean ± SD. Statistical significance was determined by one‐way ANOVA followed by Sidak's multiple comparisons test. **p < 0.01, ***p < 0.001, ns = no significance. *n* = 3–4.
**Figure S6** CD44 negative nTreg but not CD44 positive nTreg reduced macrophage‐like trans‐differentiation of VSMCs. VSMCs were cocultured with CD44 negative (CD44^−^Treg) or CD44 positive Treg (CD44^+^Treg) in the presence of Chol for 48 h. VSMCs cultured in the presence of Chol (control) were served as a control. After removing T cells gently, VSMCs from different groups were collected, the gene expression of *Il6*, *Il1b*, *Mmp2*, *Mmp3*, *Mmp12*, *Mmp13* by VSMCs from different groups was detected by qPCR. The expression level of each gene was normalized to *Gapdh* or *Actb* and calculated as fold change relative to group of control (control = 1) using the 2^‐ΔΔCt^ method. Data are presented as mean ± SD. Statistical significance was determined by one‐way ANOVA followed by Sidak's multiple comparison test. *p < 0.05, **p < 0.01, ***p < 0.001, ns = no significance. *n* = 3.
**Figure S7** The effect of iTregs on autologous T cells. ApoE^−/−^ mice were subjected to a western diet for 12 weeks and treated with CD45.1^+^ iTregs, followed by another 4 weeks of western diet before sacrificed. CD45.1, CD4, CD8a, and Foxp3 expressed by cells isolated from peri‐aortic draining lymph nodes (vLN), spleen (SP), peripheral blood (PB) and aortic tissue (vessel) was analyzed by flow cytometry. (A) The gating strategy employed during flow‐cytometric analysis is illustrated in panel A. (B) The expression of CD45.1^+^Foxp3^‐^ by CD4^+^ cells as showed in P4 by cells from different tissues of different treated mice was showed. (C) The expression of CD45.1^−^ by CD4^+^ cells as showed in P3 by cells from different tissues of different treated mice was showed. (D) The expression of CD45.1^−^Foxp3^−^ by CD4^+^ cells as showed in P5 by cells from different tissues of different treated mice was showed. Data are presented as the mean ± SD. Statistical significance was determined by one‐way ANOVA followed by Sidak's multiple comparisons test. **p < 0.01, ***p < 0.001, ns = no significance. *n* = 3–5.
**Figure S8** Effect of iTreg treatment on serum lipid content in atherosclerosis. ApoE^−/−^ mice were subjected to a western diet for 12 weeks and treated with iTreg/Med, followed by another 4 weeks of western diet before sacrificed. Serum was collected and the expression of LDL‐C, HDL‐C, total Chol (TC), and triglycerides (TG) was detected using commercial kits following the manufacturer's instructions. Data are presented as the mean ± SD. Statistical significance was determined by one‐way ANOVA followed by Sidak's multiple comparisons test. ns = no significance. *n* = 4–5.
**Figure S9** iTreg treatment reduces inflammatory cytokines in atherosclerosis. ApoE^−/−^ mice were subjected to a western diet for 12 weeks and treated with iTreg/Med, followed by another 4 weeks of western diet before sacrificed. IL‐17a, IFNγ, IL‐5, IL‐2, TNF‐α expressed by cells isolated from draining lymph node and spleen was analyzed by flow cytometry. Data are presented as the mean ± SD. Statistical significance was determined by Student's *t‐*test. *p < 0.05, ns = no significance. *n* = 7.
**Figure S10** The migration of iTregs to localized vascular regions. (A) ApoE^−/−^ mice were fed either a normal diet (ND) or a western diet (WD) for 12 weeks. Immunofluorescence staining was employed to assess the spatial relationship between CD4^+^ T cells (CD4, green) and VSMCs (ACTA2, red) in the aortic root tissue sections of both groups. (B) ApoE^−/−^ mice were subjected to a western diet for 12 weeks and then treated with iTregs or control cells (Med) derived from naive CD4^+^ T cells isolated from Foxp3^GFP^ reporter mice. After an additional 3 weeks of western diet, the mice were sacrificed and the expression of Foxp3^GFP^ in the aortic sinus sections was observed under a fluorescence microscope.
**Figure S11** Expression of LAP by iTregs. iTregs were stained with flow cytometric antibody of Foxp3/ LAP (iTreg) or Foxp3/isotype of LAP (isoType), then the expression of Foxp3 and LAP was analyzed by flow cytometry. Data are presented as the mean ±SD. Statistical significance was determined by Student's *t‐*test. ***p < 0.001, ns = no significance. *n* = 3.
**Figure S12** TGF‐β reduce macrophage‐like trans‐differentiation of VSMCs. VSMCs were stimulated with Chol and treated with TGF‐β for 48 h. VSMCs stimulated with Chol without TGF‐β treatment for 48 h is served as positive control (control), VSMCs culture alone received no stimulation or treatment for 48 h is served as a negative control (VSMC only). The expression of galectin‐3 (green) and DAPI (blue) in VSMCs was detected by immunofluorescence.
**Figure S13** Effect of iTreg treatment on VSMCs phenotype switching partly via TGF‐β signaling. VSMCs were cocultured with iTregs or not, in the presence of Chol for 48 h. In some experiments, TGF‐β signaling was blocked by adding an activin‐like kinase 5 inhibitor (ALK5i, LY3200882, 5 µM) or a TGF‐β neutralizing antibody (anti‐ TGF‐β, MA5‐23795, 1 µg/mL). VSMCs from different experimental groups were collected, and the protein expression levels of ACTA2 and MAC‐2 were analyzed by flow cytometry. Data are presented as the mean ± SD. Statistical significance was determined by two‐way ANOVA followed by multiple comparison tests. *p < 0.05, ns = no significance. *n* = 3.
**Figure S14** iTregs facilitate VSMCs apoptosis mediated by TGF‐β signaling. VSMCs were cultured in isolation (control) or cocultured with either control cells (Med) or iTregs (iTreg) in the presence of Chol for 48 h. In certain experiments involving iTreg coculture, TGF‐β signaling was inhibited by the addition of an activin‐like kinase 5 inhibitor (ALK5i, LY3200882, 5 µM). VSMCs from each treatment group were harvested and subjected to Annexin V/PI staining, followed by flow cytometric analysis. Data are presented as mean ± SD. Statistical significance was determined by one‐way ANOVA followed by Sidak's multiple comparisons. ***p < 0.001, ns = no significance. *n* = 3.
**Table S1** Murine specific primers.

## Data Availability

The datasets used and analyzed during the current study are available from the corresponding author on reasonable request.
