# Supplementary material for: Infusion of Induced Regulatory T Cells Alleviates Atherosclerosis by Reducing Pathological Macrophage‐Like Vascular Smooth Muscle Cells
Source: MedComm (2020). 2025 Oct 22;6(11):e70439. doi: 10.1002/mco2.70439 (PMC12547077; doi:10.1002/mco2.70439)
Supplement: Supplementary file 1 — Figure S1 Chol stimulation promote a macrophage‐like phenotype switching of VSMCs. VSMCs were stimulated with Chol (50 µg/mL) for 48 h to induce the transformation into a macrophage‐like phenotype. (A) The expression of macrophage‐like VSMCs was assessed by immunofluorescence staining of ACTA2 (red), MAC‐2 (green), DAPI (blue), and Oil Red O staining of foam cell formation. (B) The expression levels of VSMCs‐related marker genes Acta2, Calponin, and Tropoelastin, as well as macrophage‐related marker genes Lagls2, Lagls3, Cd68 and inflammatory cytokine genes Mcp1 and Il6 were measured by quantitative PCR (qPCR). The expression level of each gene was normalized to Gapdh or Actb and calculated as fold change relative to group of VSMCs only (VSMC only = 1) using the 2‐ΔΔCt method. Data are presented as mean ± SD. Statistical significance was determined by Student's t‐test. *p < 0.05, **p < 0.01, ns = no significance. n = 3. Figure S2 nTregs promote the phenotypic conversion of VSMCs. VSMCs were cocultured with nTregs in the presence of Chol for 48 h (Chol + VSMC + nTreg), VSMCs cocultured with nTregs with no Chol (VSMC + nTreg) or VSMCs cultured alone without (VSMC only) or with (VSMC + Chol) Chol stimulation were used controls. Expression of ACTA2 and MAC‐3 by VSMCs from different groups was analyzed by flow cytometry. Data are presented as the mean ± SD. Statistical significance was determined by one‐way ANOVA followed by Sidak's multiple comparisons test. *p < 0.05, **p < 0.01, ***p < 0.001, ns = no significance. n = 3. Figure S3 Phenotypic conversion of VSMCs in atherosclerosis. ApoE−/− mice were subjected to a western diet for 12 weeks to 20 weeks, expression of ACTA2 (red), CD68 (green), and DAPI (blue) in the aortic root was detected by immunofluorescence staining. Figure S4 The balance of immune cells in atherosclerosis was disrupted. ApoE−/− mice were subjected to a normal diet (ND) for 5 months or a western diet for 3–5 months, the expression of CD44, CD62L, C [file MCO2-6-e70439-s001.docx]

**Supplementary Materials for**

**Infusion of induced regulatory T cells alleviates atherosclerosis by reducing pathological macrophage-like** **vascular smooth muscle cells**

**Running Title:** **iTregs and phenotypic switching of VSMCs in atherosclerosis**

Ximei Zhang^1#^, Ye Chen^2#^, Yesheng Ling^1#^, Lin Wu^1^, Dinghui Liu^1^, Linli Wang^1^, Yong Liu^1^, Guangyao Shi^1^, Bin Zhou^1^, Baoshun Hao^1^, Zhenda Zheng^1^, Shujie Yu^1^, Min Wang^1^, Jun Zhao^3^, Donglan Zeng^3^, Julie Wang^2^, Yan Lu^3^, Jun Tao^4^, Wenhao Xia^4^, Song Guo Zheng^2^*, Xiaoxian Qian^1^*

**Affiliations**

^1^Department of Cardiology, Third Affiliated Hospital of Sun Yat-sen University, Guangzhou 510630, China.

^2^Department of Immunology, School of Cell and Gene Therapy, Songjiang Research Institute and Songjiang Hospital Affiliated to the Shanghai Jiaotong University School of Medicine, Shanghai 201600, China.

^3^Department of Clinical Immunology, Third Affiliated Hospital of Sun Yat-sen University, Guangzhou 510630, China.

^4^Department of Hypertension and vascular disease, First Affiliated Hospital of Sun Yat-sen University, Guangzhou 510080, China.

^#^These authors contributed equally to this work.

*Senior authors contribute equally.

*Corresponding author, e-mail address: Song.Zheng@shsmu.edu.cn and [qianxx@mail.sysu.edu.cn](mailto:qianxx@mail.sysu.edu.cn).

**This PDF file includes:**

**Materials and Methods**

**Figure S1 to S14**

**Materials and Methods**

**Mice**

Mice were purchased from Beijing Vital River Laboratory Animal Technology Co., Ltd (Beijing, China). The mice were bred and maintained at the Guangdong Provincial Laboratory Animal Monitoring Institute under specific pathogen-free and standard laboratory conditions. All animal procedures were performed in compliance with the procedures approved by the Animal Welfare Ethics Committee of the Guangdong Provincial Department of Science and Technology. To exclude possible hormonal effects (e.g., estrogen), only male mice were used in the experiments.

**Atherosclerosis and histopathology**

Eight-week-old male Apolipoprotein E knock out (ApoE^-/-^) mice were randomly distributed to each group and fed a WD with 1.25% cholesterol and 40% (w/w) fat (D12108C; Research Diets) for 15 weeks to induce atherosclerosis. For iTreg treatment, iTregs were induced as previously reported[^80^](#_ENREF_80)^,^[^81^](#_ENREF_81). Briefly, 2.0x10^6^ iTregs suspended in 200 μl sterile phosphate-buffered saline (PBS) were injected intravenously after 12 weeks of WD, followed by another 3 weeks of WD before sacrifice. The mice treated with PBS only or control cells were used as the control or vehicle control, respectively. To block TGF-β signaling, TGF-β receptors type I/II inhibitor (LY2109761, 0.5 mg/mouse) was administered intraperitoneally every 3 days, starting immediately after iTreg injection and ending at the point of sacrifice. After 15 weeks of WD, the mice were sacrificed by CO_2_ gas inhalation, and blood was obtained through cardiac puncture to measure serum lipid concentrations. After successively opening the abdominal and thoracic cavities, the mice were perfused with pre-cooled PBS through the heart to eliminate blood. Then, inguinal [lymph nodes](javascript:;), spleens, peri-aortic lymph nodes, hearts and whole aortas were collected. After peri-adventitial tissue removal, the aortas were opened longitudinally and soaked in DMEM supplemented with 2% fetal bovine serum (FBS) for further single cell isolation or Oil Red O staining. The aortic root and cardiac base were embedded in optimal cutting temperature (OCT) medium, snap-oriented horizontally on dry ice until complete solidification, and subsequently stored at −80 °C pending cryosectioning. The cryosections (5μm or 10μm) perpendicular to the aortic sinus were made sequentially every 50μm. From this, approximately 10-15 serial sections spanning 500-750μm of the aortic sinus were made. After handling the mice, the investigators were blinded when assessing the outcome.

For Oil Red O staining, 0.5g of Oil Red O dry powder (Sigma-Aldrich, O0625) was dissolved in 100 ml of 100% isopropanol to obtain a storage solution. Before staining, the storage solution was diluted with ddH_2_O at a ratio of 3:2 and filtered to make a fresh working solution. Slides were fixed with methanol and stained with the working solution for 10 min at room temperature. After differentiating in 60% isopropanol for 30s, the slides were washed with running water and mounted with glycerol. Hematoxylin and eosin staining (Solarbio, G1120) and Masson’s trichrome staining (Solarbio, G1340) were performed using commercial kits following the manufacturer’s instructions.

**Characterization of aortic leukocytes and VSMCs**

The whole aorta, from the ascending aorta to the bifurcation of the common iliac artery, was opened longitudinally and minced into 1-2mm pieces in RPMI culture medium supplemented with 2% FBS. To characterize aortic leukocytes in aortic tissues, aortic pieces were resuspended in a fresh enzyme cocktail with collagenase type I (Sigma-Aldrich, C0130, 675 U/ml), collagenase type XI (Sigma-Aldrich, C7657, 187.5 U/ml), and hyaluronidase type I-s (Sigma-Aldrich, H1115000, 9 U/ml) and incubated in a shaker at 37°C for 45min. Following incubation, the samples were filtered through a 70-μm strainer and centrifuged at 300g for 5min. The cell pellets were washed once with FACS buffer (2% FBS in PBS), stained with Fixable Viability Dye eFluor^TM^ 780 (eBioscience, #65-0865-14) as well as fluorochrome-conjugated antibodies specific for mouse such as CD3, CD4, CD8, B220, CD62L, CD44, CD11b, Ly6C, Ly6G, CD45.1, Foxp3, IFN-γ, and IL-17a, and analyzed by flow cytometry[^82^](#_ENREF_82). For characterization of aortic VSMCs, aortic pieces were resuspended in an enzymatic solution containing 2 mg/ml of collagenase II (Sigma, C6885) in DMEM culture medium supplemented with 2% FBS, and incubated in a shaker at 37°C for 30-45min. Following incubation, the samples were filtered through a 70μm strainer and centrifuged at 300g for 5min. The cell pellets were washed once with FACS buffer (2% FBS in PBS), stained with fluorochrome-conjugated antibodies specific for mouse ACTA2, CD68, MAC-2, and MAC-3, and analyzed by flow cytometry[^83^](#_ENREF_83).

**Immunofluorescence staining**

Frozen sections were fixed in methanol for 15min, washed three times with PBS, blocked with 10% goat serum at RT for 1h, and incubated with primary antibodies, including rabbit anti-ACTA2 (1:200, 14395-1-AP, Proteintech), FITC anti-mouse CD4 (1:200, 100406, Biolegend), rat anti-CD68 (1:400, Abcam, ab53444), mouse anti-MAC-3(1:200, 66301-1-Ig, Proteintech) or mouse anti-MAC-2 (1:200, 60207-1-Ig, Proteintech) at 4°C overnight. Sections were washed three times with PBS and then incubated with secondary antibodies, including goat anti-rabbit IgG H&L (Alexa Fluor® 568) (1:1000, Abcam, ab175471) goat anti-mouse IgG H&L (Alexa Fluor® 488) (1:500, Invitrogen, A11001), and [goat anti-rat IgG H&L (Alexa Fluor® 488) (1:1000, Abcam, ab150157)](https://www.abcam.cn/products/secondary-antibodies/goat-rat-igg-hl-alexa-fluor-488-ab150157.html) for 1h at RT. After washing with PBS three times, the sections were mounted with glycerol.

**Naïve CD4^+^ T cell and iTreg**

The peripheral lymph nodes and spleens were collected from C57BL/6 mice and ground to obtain single cells, before eliminating splenic erythrocytes using red blood cell lysis buffer (Sigma-Aldrich). Total T cells were enriched with nylon wool, followed by magnetic cell sorting with an auto magnetic cell sorter (MACS) (MiltenyiBiotec, Germany) to obtain purified naïve CD4^+^ T cells. In brief, enriched T cells labeled with biotin anti-CD8, CD25, B220, CD11b, CD11C, and CD49b antibodies and anti-biotin microbeads were subjected to depletion, followed by positive selection with CD62L microbeads by auto MACS. The purity of naïve CD4^+^ T cells was determined as CD4^+^CD25^+^CD62L^+^ using flow cytometry, and naïve CD4^+^ T cells with purity >95% were used to induce iTregs or control cells (Med).

For iTreg induction, purified naïve CD4^+^ T cells were cultured in the presence of anti-CD3/CD28-coated beads (cells to beads at a 1:5 ratio), rhIL-2 (50 U/ml), and rhTGF-β (2 ng/ml) in a 48-well plate for 3 days to induce iTregs as previously described[^84^](#_ENREF_84). Cells cultured under a similar condition except the presence of rhTGF-β served as control cells (Medium, Med). The percentage of Foxp3 expression was detected by flow cytometry and was found to be approximately 50% for iTregs and 10% for control cells.

For naïve CD4^+^ T cells isolated from Foxp3^GFP^ C57BL/6 mice, iTregs were collected and labeled with fluorochrome-conjugated antibodies specific for mouse CD4 antibodies, CD4^+^Foxp3^+^ T cells were sorted by flow cytometry and used as purified iTregs. The purity of purified iTreg was determined as the expression percentage of CD4^+^ Foxp3^+^ by flow cytometry and was approximately 95%.

**nTreg isolation**

For isolation of nTregs, enriched T cells obtained from Foxp3^GFP^ C57BL/6 mice as showed above were labeled with fluorochrome-conjugated antibodies specific for mouse CD4 antibodies and CD4^+^Foxp3^+^ T cells were sorted by flow cytometry and used as nTregs. The purity of the nTregs was determined as the expression percentage of CD4^+^ Foxp3^+^ by flow cytometry and was approximately 95%.

**Treg co-culture with macrophage-like VSMCs**

VSMCs were treated with water-soluble cholesterol (Chol, catalog no. C4951, 10μg/ml) for 72h to induce macrophage-like VSMCs phenotypic switching, as previously reported[^85^](#_ENREF_85). For co-culture, VSMCs were pre-treated with water-soluble cholesterol for 24h, and then co-cultured with Tregs (Tregs: VSMCs=10:1) or control cells with or without the presence of water-soluble cholesterol. After 72h of co-culture, T cells and treated VSMCs were collected separately and labeled with different target proteins, followed by analyzing with flow cytometry.

**Flow cytometry staining and analysis**

Flow cytometry staining was performed as previously reported[^38^](#_ENREF_38). For cell surface antigen staining, flow cytometry antibodies related to different antigens were diluted with PBS at 1:200, and the cells were re-suspended in 100μl of this dilution and stained for 15min in the dark at 4°C. Subsequently, the cells were washed twice with PBS and resuspendedin300-500 μl of PBS, before immediately detecting the expression of related markers by flow cytometry. For intranuclear Foxp3 staining, the eBioscience™ Foxp3/Transcription Factor Staining Buffer Set (ThermoFisher, 00-5523-00) was used, and staining was performed as described in the protocol provided with the kit. For intracellular cytokine staining, cells were stimulated with PMA (0.05 μg/ml) and ionomycin (0.5 μg/ml) for 1h, followed by the addition of brefeldin A (10 μg/ml) and incubating for another 4h at 37°C in a humidified incubator with 5% CO_2_. After stimulation, cell surface antigen staining was performed first, before fixing the cells with fixation buffer (BioLegend, 420801) for 30min at room temperature in the dark, followed by permeabilization with 1x Intracellular Staining Perm Wash Buffer (BioLegend, 421002) for another 15min. Target flow cytometric antibodies were diluted with 1x Intracellular Staining Perm Wash Buffer at 1:100 and the fixed/permeabilized cells were then resuspended with 100 μl of this dilution and stained overnight. For intra-nuclear and intra-cellular staining together, the protocol for intra-nuclear staining was followed. After staining, the cells were washed with PBS at least twice, resuspended in 300-500μl PBS, and analyzed by flow cytometry.

**Quantitative real-time PCR**

Total RNA was extracted from cultured VSMCs or mouse aortas using TRIzol reagent according to the manufacturer’s protocol. The RNA concentration, quality, and purity were determined using a Nanodrop spectrophotometer (Thermo Fisher Scientific). Complementary DNA (cDNA) was synthesized from 200-1,000 ng of total RNA using a Fast Reverse Transcription Kit (ESscience, RT001). Then, quantitative PCR (qPCR) was performed with specific gene primers and 2x Super SYBR Green qPCR Master Mix (ESscience, QP002) using the QuantStudio 7 Flex Real-Time PCR System (Applied Biosystems). Primers used in the study are listed in Table S1.

**Isolation and culture of primary VSMCs**

Primary murine VSMCs were isolated from mouse aortas and were routinely maintained in DMEM supplemented with 10% serum (FBS) as described in a previous study[^83^](#_ENREF_83). Briefly, aortas were obtained from 6-8 weeks old WT (C57BL6/J) mice. For adventitia removal, the aortas were incubated for 5min in enzymatic solutions containing 2 mg/ml of collagenase II (Sigma, C6885) in DMEM culture medium supplemented with 2% FBS. The adventitia was then removed in a similar manner to taking off a sock. Furthermore, the aortas were cut open longitudinally using small scissors, and the endothelium was removed by gentle scraping. The medial layer was minced into small pieces and fully digested into single cells by incubating via the same enzymatic method used for adventitia removal for 30min. After filtering with a Cell Strainer (70μm), the single-cell digestion solution was centrifuged to remove the digestion solution. The cells were washed twice with DMEM supplemented with 2% FBS, resuspended in VSMCs culture medium containing DMEM, 10% FBS, and 1% penicillin/streptomycin, transferred into a culture dish, and incubated in a 5% CO_2_ atmosphere at 37°C. VSMCs were analyzed by flow cytometry following staining for a smooth muscle cell (SMC) marker using a mouse monoclonal anti-Actin, α-Smooth Muscle-FITC antibody (Sigma-Aldrich, F3777), to ensure that the purity of VSMCs was above 95%. VSMCs at passages 3-5 were used for experiments.

**Migration assay**

Migration of VSMCs was determined by a scratch wound healing assay as reported previously[^86^](#_ENREF_86). Briefly, 2.5x10^5^ cells/well (three replicates per group) were plated into a 24-well plate and grown to confluence. The monolayer was scratched using a 200μl pipette tip and washed gently with a DMEM medium supplemented with 2% FBS to remove detached cells. The cells were then co-cultured with or without iTregs in the presence of water-soluble cholesterol. After 24 h of co-culture, the suspended T cells were collected gently using a 2% FBS in DMEM, leaving adherent VSMCs in the culture plates. Then the VSMCs were fixed with 4% paraformaldehyde for 20min, followed by staining with 1% crystal violet in ddH_2_O for 15min. Finally, the healing of the scratch wound was observed under a bright field microscope.

**Apoptosis assay**

2.5x10^5^ cells/well (three replicates per group) were plated into a 24-well plate and grown to confluence and then co-cultured with or without iTregs in the presence of water-soluble cholesterol. After 24 h of co-culture, the suspended T cells were removed gently, leaving adherent VSMCs in the culture plates. Then the VSMCs were collected and subjected to apoptosis analyses using an Annexin V-FITC Apoptosis Detection Kit (Beyotime, C1062M) according to the manufacturer’s instructions. After staining, the cells were analyzed by flow cytometry. Cells that stained as Annexin V^−^/propidium iodide (PI)^-^, Annexin V^+^/PI^-^, Annexin V^+^/PI^+^, or Annexin V^-^/PI^+^ were counted as live, early apoptotic, late apoptotic, or dead/necrotic cells, respectively.

**Statistical analysis**

The data are presented as means ± standard deviation (SD) and were analyzed utilizing GraphPad Prism (GraphPad Software, San Diego, CA, USA). Statistical analyses and comparisons were conducted using Student's *t*-test, one-way analysis of variance (ANOVA), and for comparisons between two groups under different conditions, two-way ANOVA was employed. A p-value of less than 0.05 was deemed statistically significant.

**Figure S1**

**
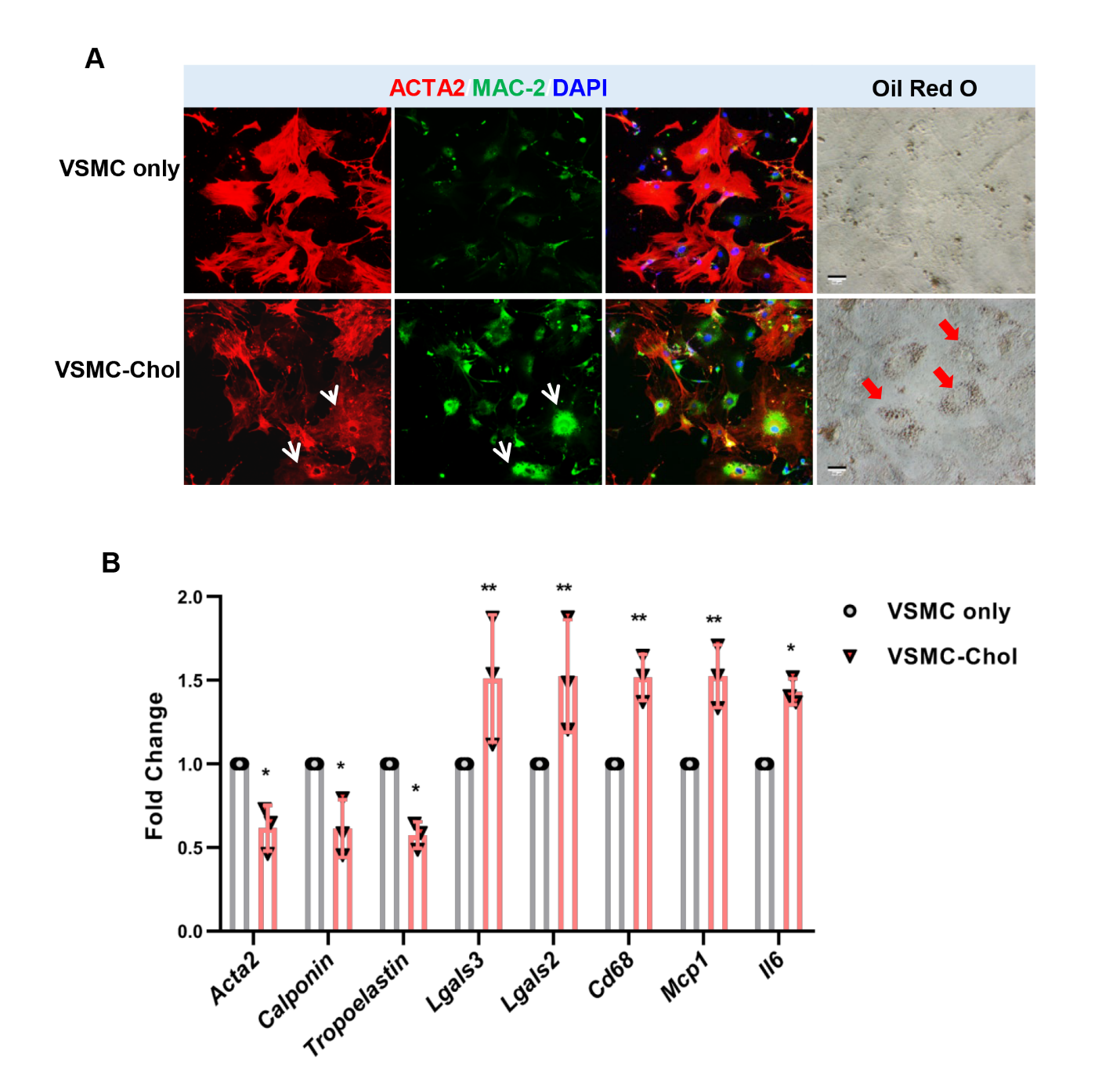
Figure S1 Cholesterol stimulation promote a Macrophage-like phenotype switching of VSMCs.** VSMCs were stimulated with cholesterol (50 µg/mL) for 48 hours to induce the transformation into a macrophage-like phenotype. (A) The expression of macrophage-like VSMCs was assessed by immunofluorescence staining of ACTA2 (Red), MAC-2 (Green), DAPI (Blue) and Oil Red O staining of foam cell formation. (B) The expression levels of VSMCs-related marker genes *Acta2*, *Calponin*, and *Tropoelastin*, as well as macrophage-related marker genes *Lagls2*, *Lagls3*, *Cd68* and inflammatory cytokine genes *Mcp1* and *Il6* were measured by quantitative PCR (qPCR). The expression level of each gene was normalized to *Gapdh* or *Actb* and calculated as fold change relative to group of VSMCs only (VSMC only = 1) using the 2^-ΔΔCt^ method. Data are presented as mean ± SD. Statistical significance was determined by Student's t-test. * *P* < 0.05, ** *P* < 0.01, ns=no significance. n= 3.

**Figure S2**

**
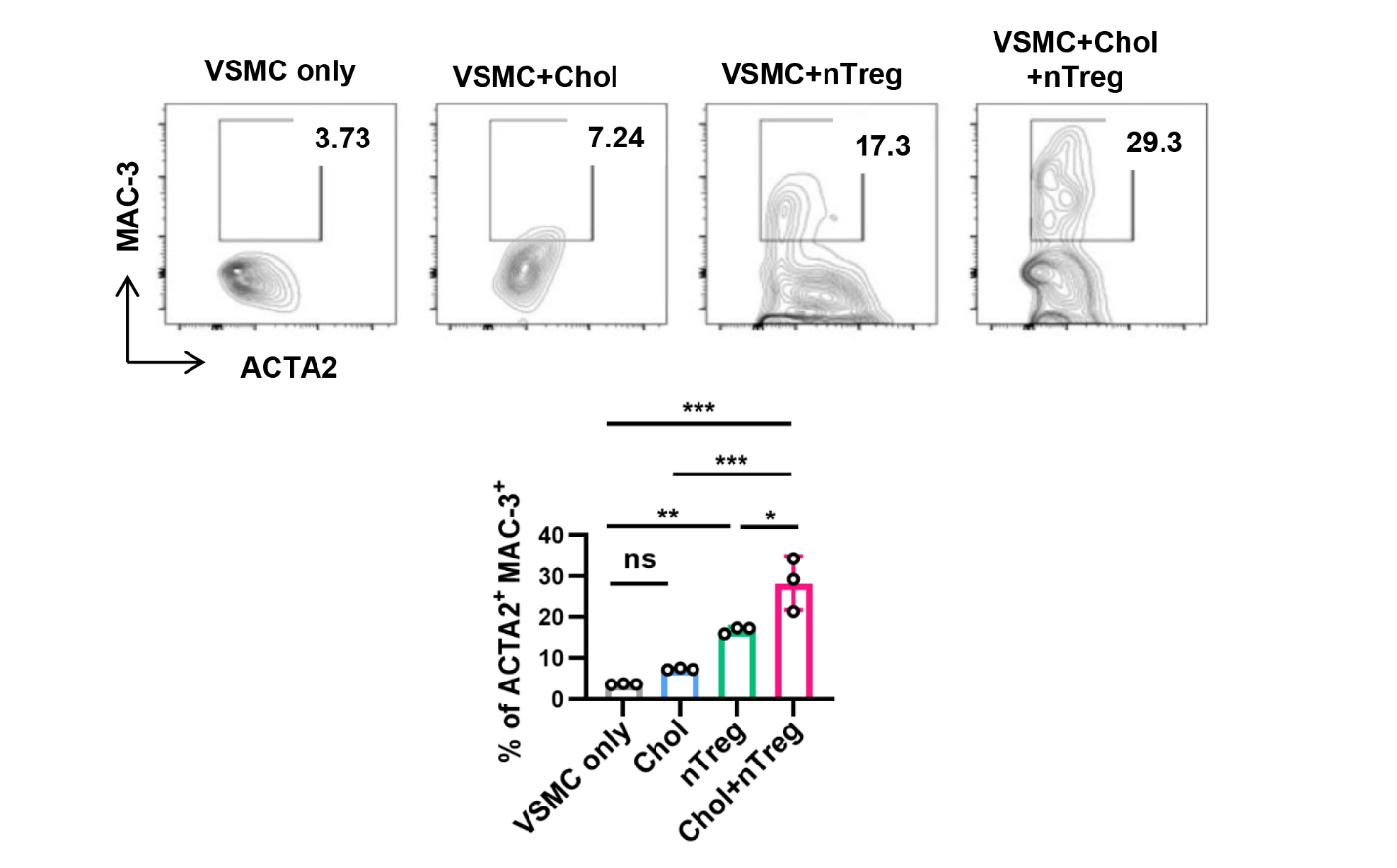
**

**Figure S2 nTregs promote the phenotypic conversion of VSMCs.** VSMCs were co-cultured with nTregs in the presence of cholesterol for 48h (Chol+VSMC+nTreg), VSMCs co-cultured with nTregs with no cholesterol (VSMC+nTreg) or VSMCs cultured alone without (VSMC only) or with (VSMC+Chol) cholesterol stimulation were used controls. Expression of ACTA2 and MAC-3 by VSMCs from different groups was analyzed by flow cytometry. Data are presented as the mean ± SD. Statistical significance was determined by One-way ANOVA followed by Sidak’s multiple comparisons test. * *P* < 0.05, ** *P* < 0.01, *** *P* < 0.001, ns=no significance. n=3.

**Figure S3**


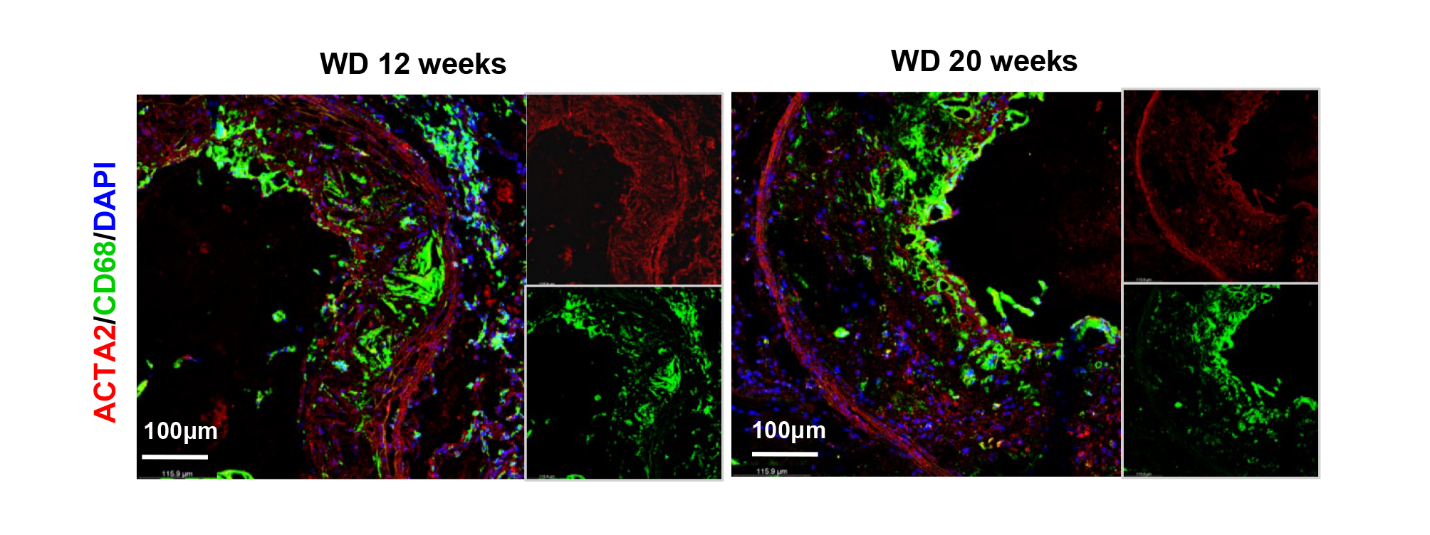


**Figure S3 Phenotypic conversion of VSMCs in atherosclerosis.**

ApoE^-/-^ mice were subjected to a western diet for 12 weeks to 20 weeks, expression of ACTA2 (Red), CD68 (Green) and DAPI (Blue) in the aortic root was detected by immunofluorescence staining.

**Figure S4**


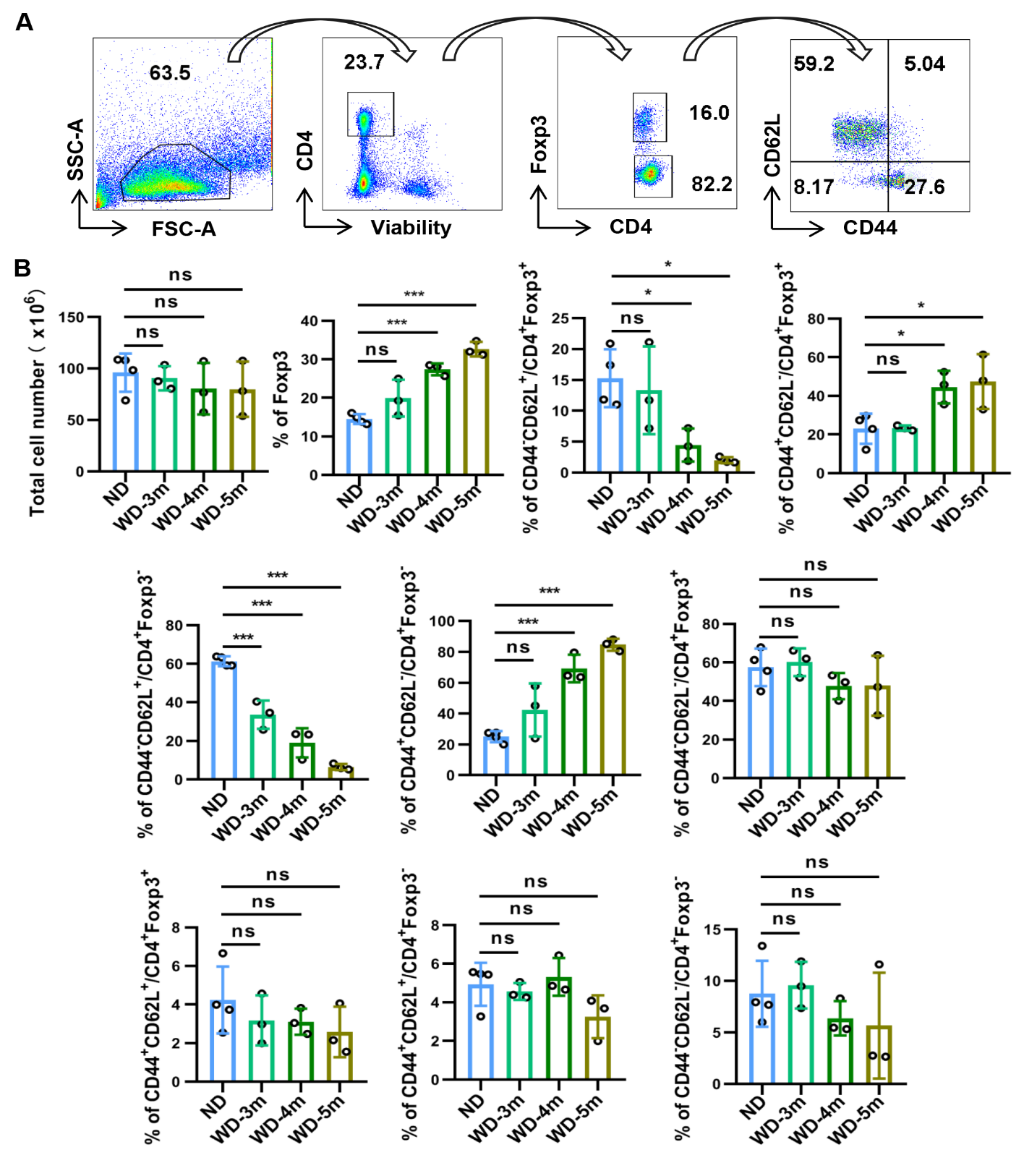


**Figure S4 The balance of immune cells in atherosclerosis was disrupted.**

ApoE^-/-^ mice were subjected to a Normal diet (ND) for 5 months or a western diet for 3-5 months, the expression of CD44, CD62L, CD4 and Foxp3 in cells isolated from spleens were detected by Flow Cytometry. **(A)** The gating strategy employed during flow cytometric analysis is illustrated in panel **A**. **(B)** Data in the bar graphs are presented as the mean ± SD. Statistical significance was determined by One-way ANOVA followed by Sidak’s multiple comparisons test. **P* < 0.05, *** *P* < 0.001, ns=no significance. n=3-4.

**Figure S5**

**
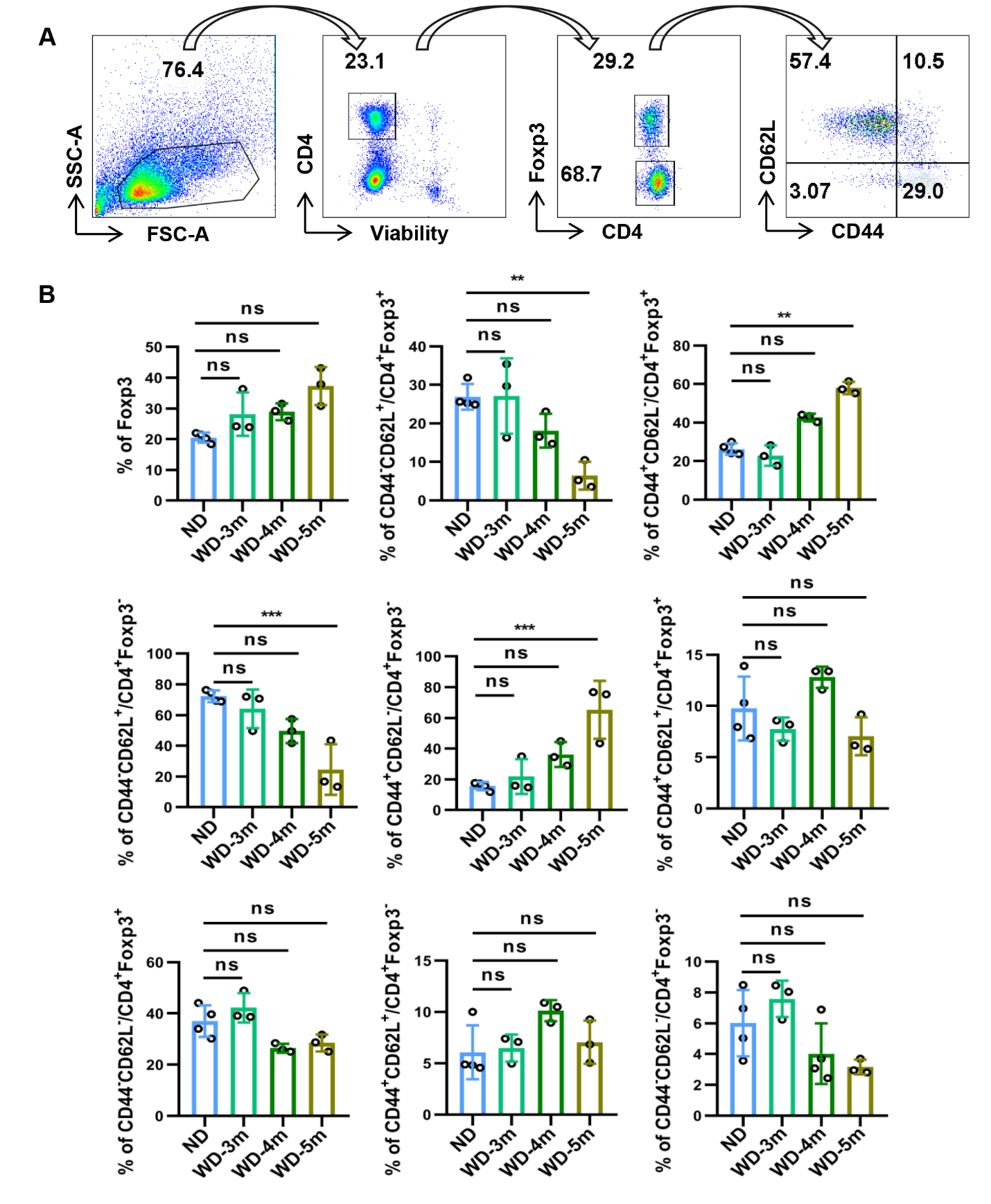
**

**Figure S5 The balance of immune cells in atherosclerosis was disrupted.**

ApoE^-/-^ mice were subjected to a Normal diet (ND) for 5 months or a western diet for 3-5 months, the expression of CD44, CD62L, CD4 and Foxp3 in cells isolated from peri-aortic lymph nodes were detected by Flow Cytometry. **(A)** The gating strategy employed during flow-cytometric analysis is illustrated in panel **A**. **(B)** Data in the bar graphs are presented as the mean ± SD. Statistical significance was determined by One-way ANOVA followed by Sidak’s multiple comparisons test. ** *P* < 0.01, *** *P* < 0.001, ns=no significance. n=3-4.

**Figure S6**


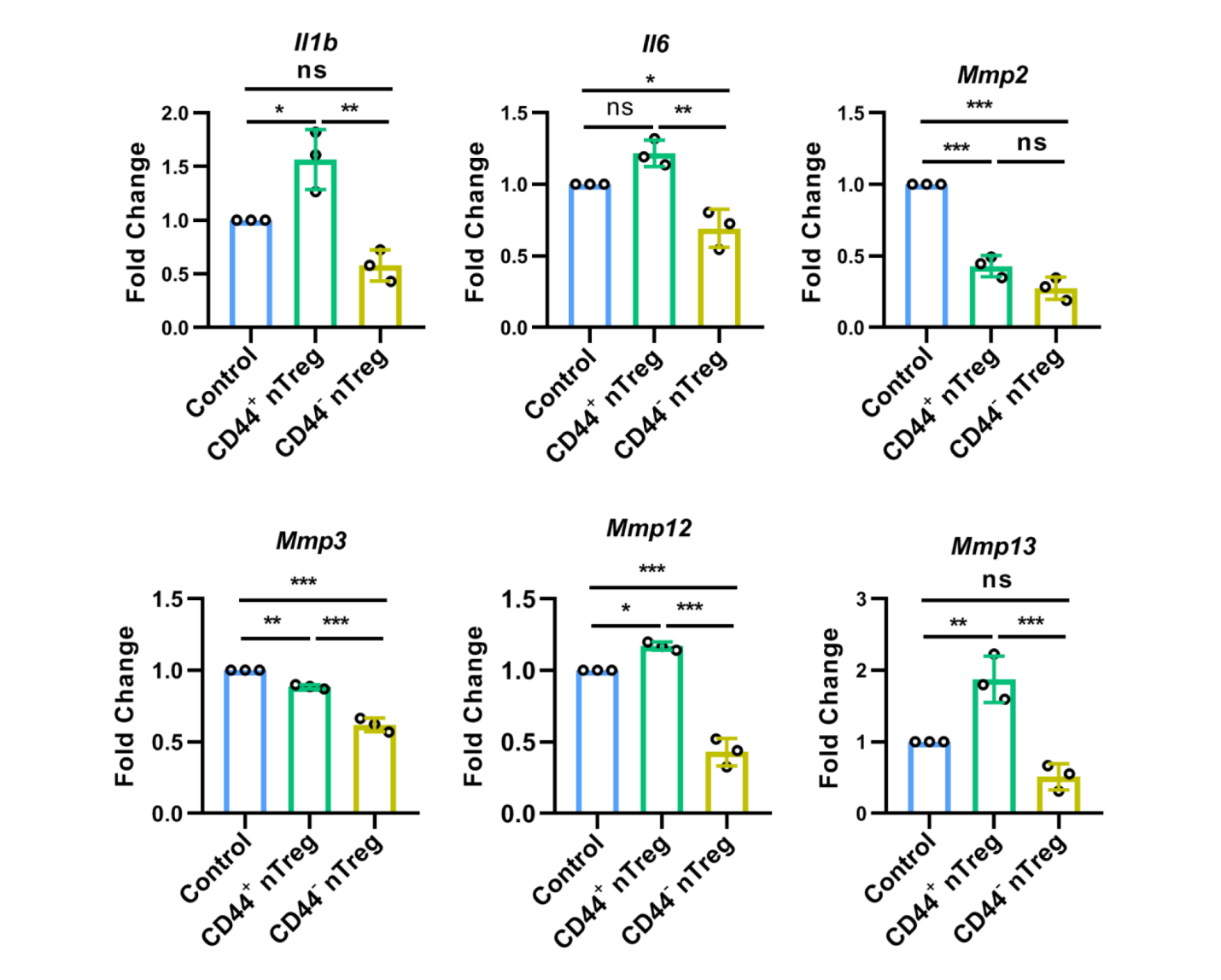


**Figure S6 CD44 negative nTreg but not** **CD44 positive nTreg reduced macrophage-like trans-differentiation of VSMCs.**

VSMCs were co-cultured with CD44 negative (CD44^-^Treg) or CD44 positive Treg (CD44^+^Treg) in the presence of cholesterol for 48h. VSMCs cultured in the presence of cholesterol (Control)were served as a control. After removing T cells gently, VSMCs from different groups were collected, the gene expression of *Il6, Il1b, Mmp2, Mmp3, Mmp12, Mmp13* by VSMCs from different groups was detected by qPCR. The expression level of each gene was normalized to *Gapdh* or *Actb* and calculated as fold change relative to group of control (control = 1) using the 2^-ΔΔCt^ method. Data are presented as mean ± SD. Statistical significance was determined by One-way ANOVA followed by Sidak’s multiple comparison test. **P* < 0.05, ***P* < 0.01, ****P* < 0.001, ns=no significance. n = 3.

**Figure S7**

**
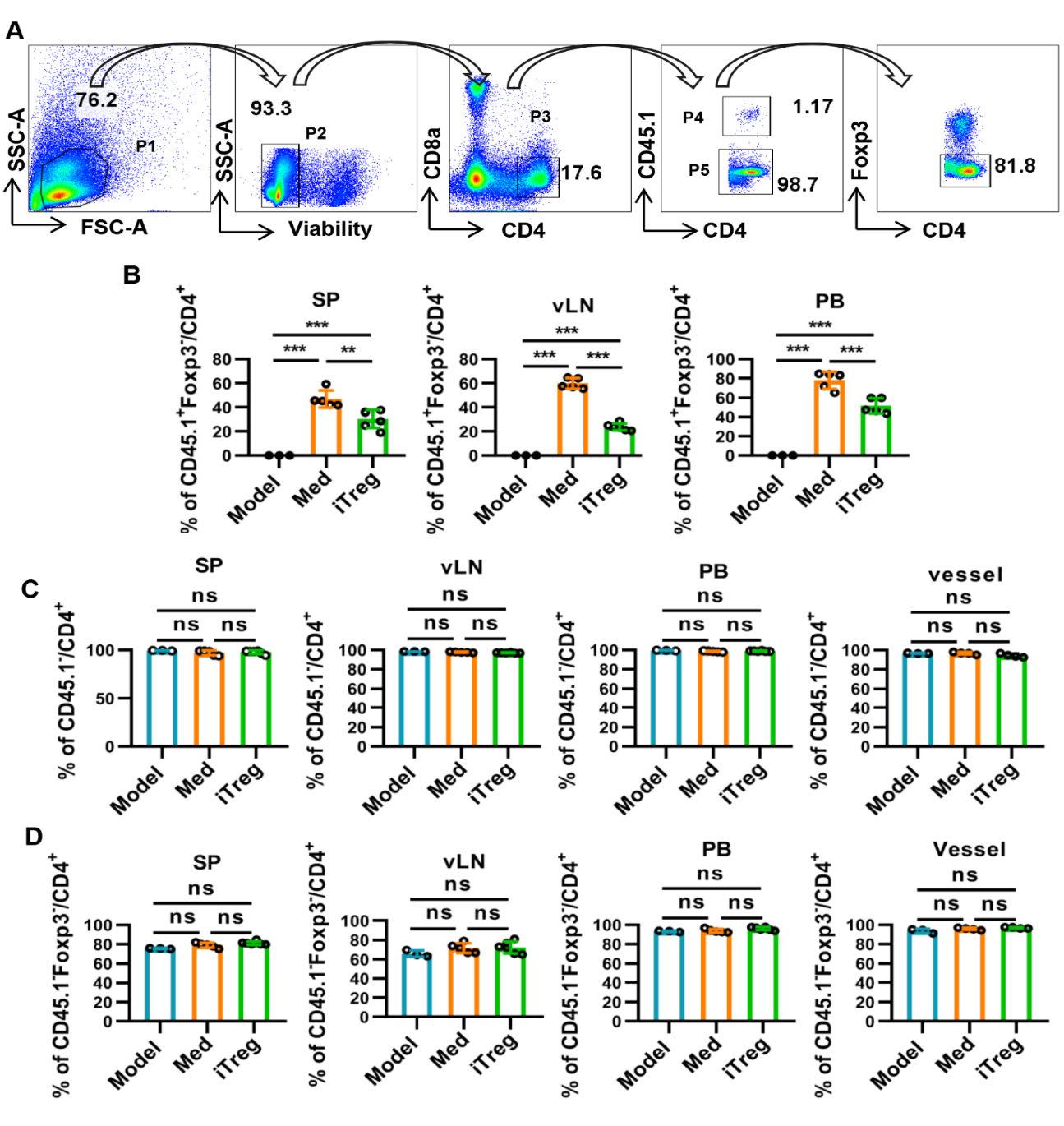
**

**Figure S7 The effect of iTregs on autologous T cells.**

ApoE^-/-^mice were subjected to a western diet for 12 weeks and treated with CD45.1^+^ iTregs, followed by another 4 weeks of western diet before sacrificed. CD45.1, CD4, CD8a and Foxp3 expressed by cells isolated from peri-aortic draining lymph nodes (vLN), spleen (SP), peripheral blood (PB) and aortic tissue (Vessel) was analyzed by flow cytometry. (A) The gating strategy employed during flow-cytometric analysis is illustrated in panel **A**. (B) The expression of CD45.1^+^Foxp3^-^ by CD4^+^ cells as showed in P4 by cells from different tissues of different treated mice was showed. (C) The expression of CD45.1^-^ by CD4^+^ cells as showed in P3 by cells from different tissues of different treated mice was showed. (D) The expression of CD45.1^-^Foxp3^-^ by CD4^+^ cells as showed in P5 by cells from different tissues of different treated mice was showed. Data are presented as the mean ±SD. Statistical significance was determined by One-way ANOVA followed by Sidak’s multiple comparisons test. ***P* < 0.01, ****P* < 0.001, ns=no significance. n=3-5.

**Figure S8**


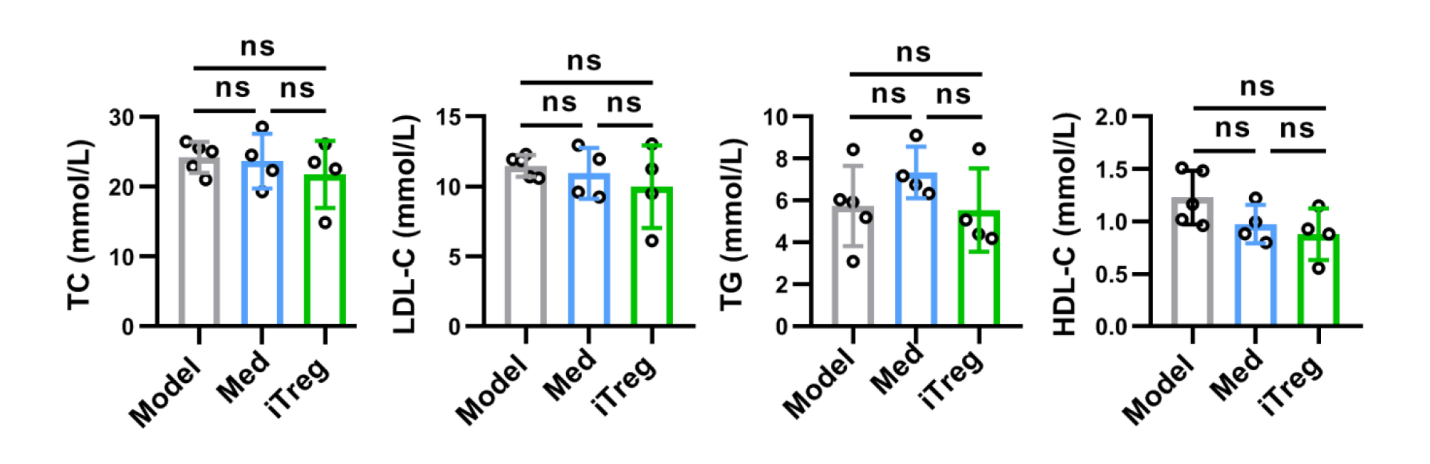


**Figure S8 Effect of iTreg treatment on serum lipid content in atherosclerosis.** ApoE^-/-^ mice were subjected to a western diet for 12 weeks and treated with iTreg/Med, followed by another 4 weeks of western diet before sacrificed. Serum was collected and the expression of LDL-C, HDL-C, total cholesterol (TC) and triglycerides (TG) was detected using commercial kits following the manufacturer’s instructions. Data are presented as the mean ±SD. Statistical significance was determined by One-way ANOVA followed by Sidak’s multiple comparisons test. ns=no significance. n=4-5.

**Figure S9**


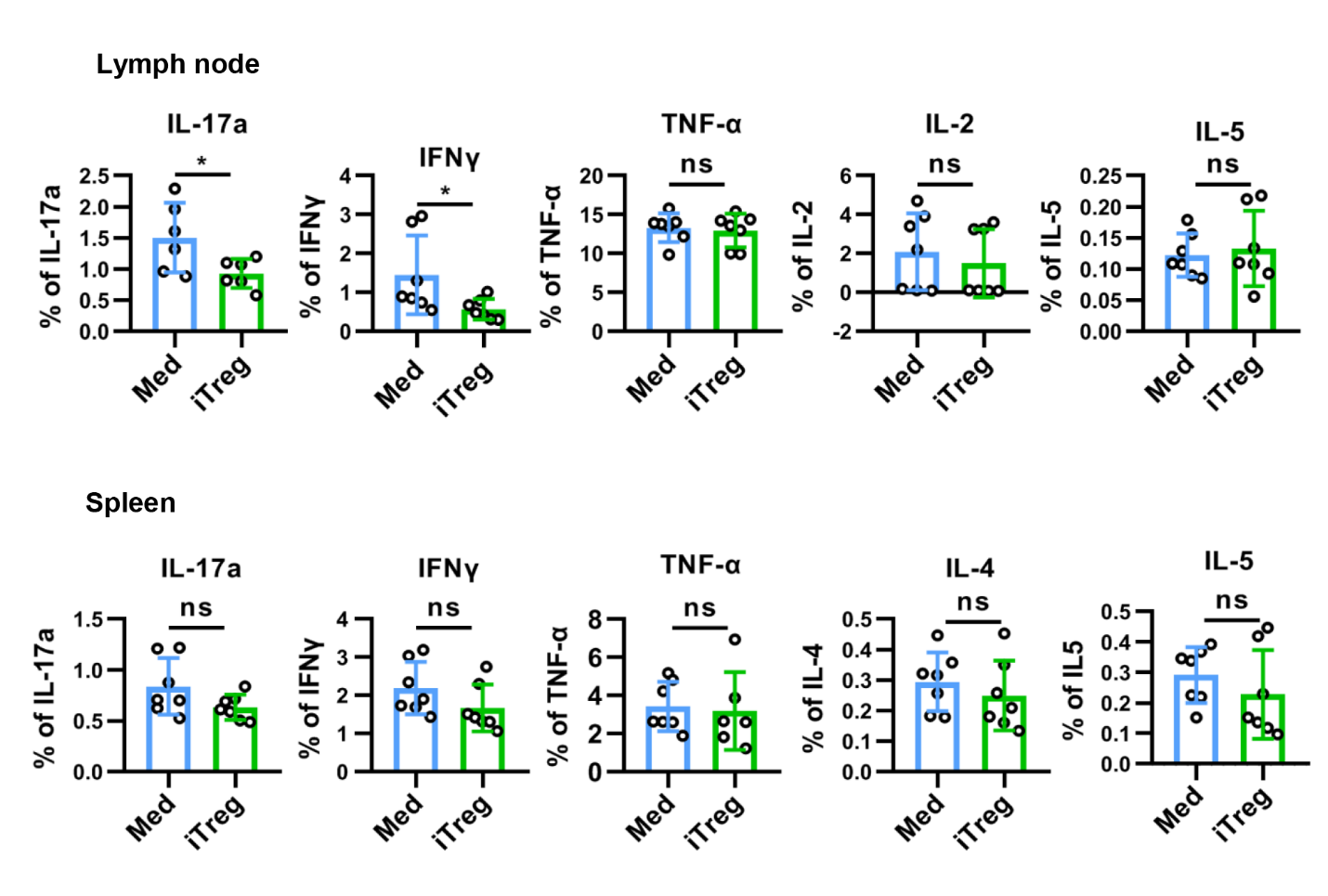


**Figure S9 iTreg treatment reduces inflammtory cytokines in atherosclerosis.** ApoE^-/-^ mice were subjected to a western diet for 12 weeks and treated with iTreg/Med, followed by another 4 weeks of western diet before sacrificed. IL-17a, IFNγ, IL-5, IL-2, TNF-α expressed by cells isolated from draining lymph node and spleen was analyzed by flow cytometry. Data are presented as the mean ±SD. Statistical significance was determined by Student's *t* test. **P* < 0.05, ns=no significance. n=7.

**Figure S10**

**
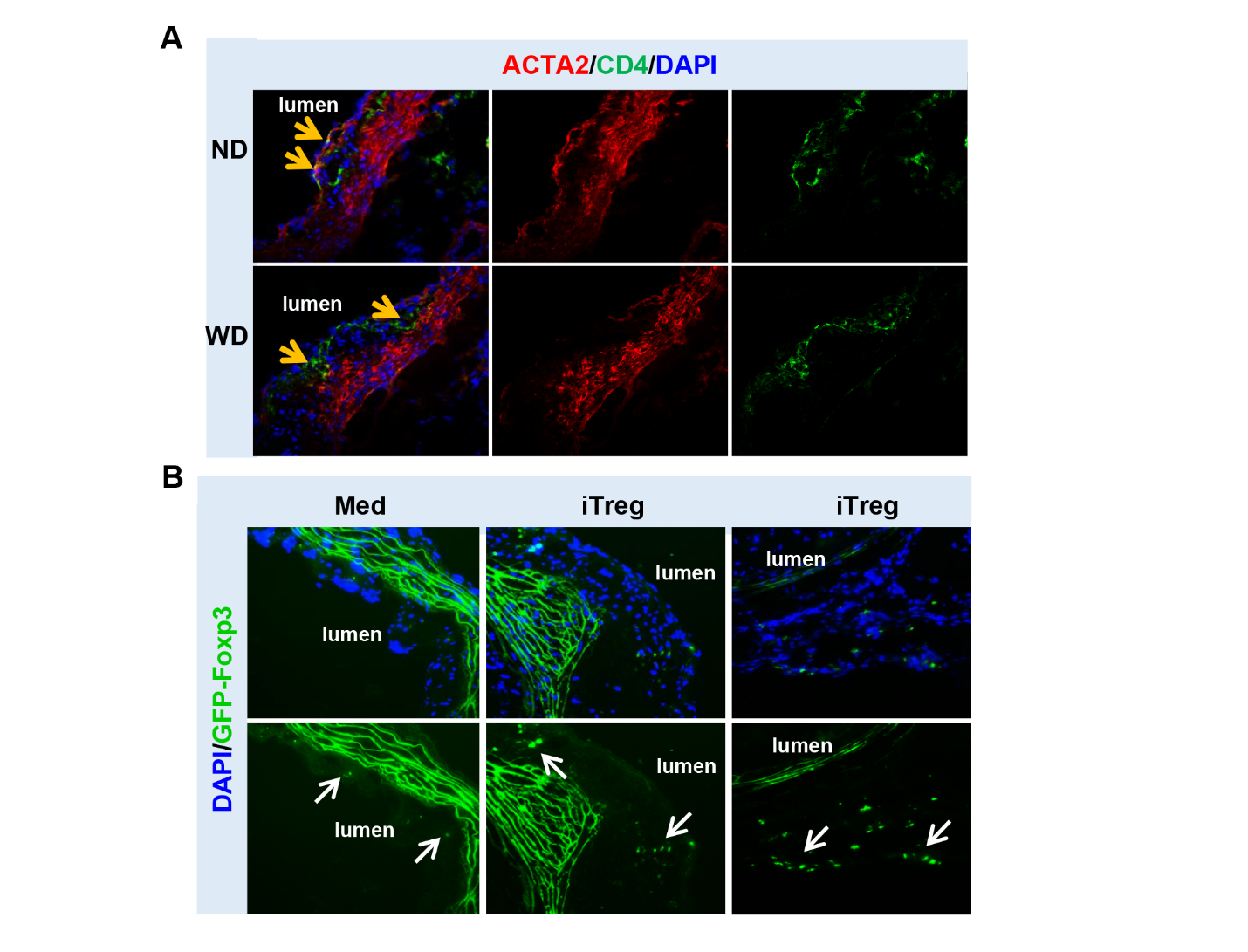
**

**Figure S10 The migration of iTregs to localized vascular regions**

**(A)** ApoE^-/-^ mice were fed either a normal diet (ND) or a western diet (WD) for 12 weeks. Immunofluorescence staining was employed to assess the spatial relationship between CD4^+^ T cells (CD4, green) and VSMCs (ACTA2, red) in the aortic root tissue sections of both groups. **(B)** ApoE^-/-^ mice were subjected to a western diet for 12 weeks and then treated with iTregs or control cells (Med) derived from naive CD4^+^ T cells isolated from Foxp3^GFP^ reporter mice. After an additional 3 weeks of western diet, the mice were sacrificed and the expression of Foxp3^GFP^ in the aortic sinus sections was observed under a fluorescence microscope.

**Figure S11**

**
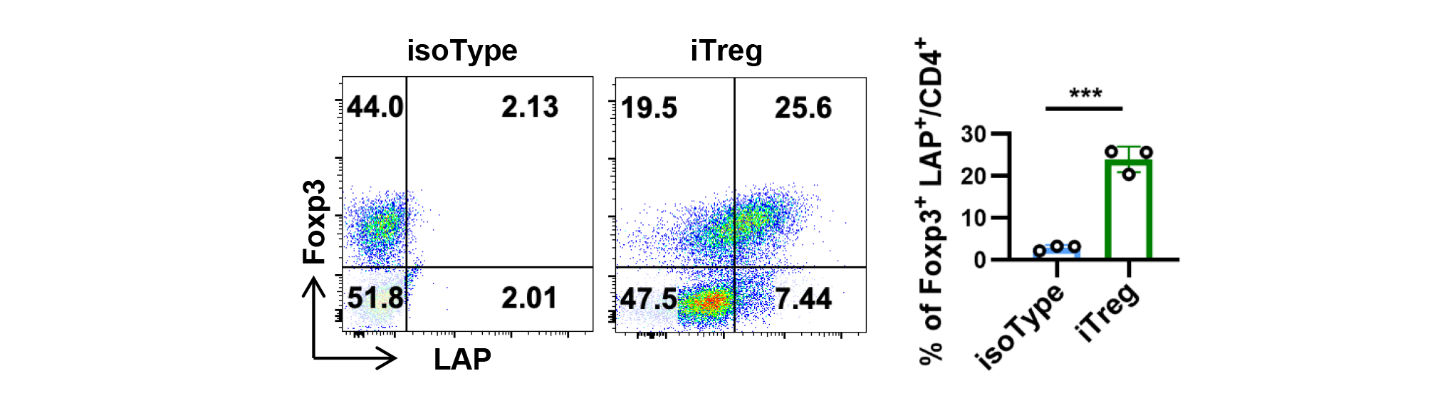
**

**Figure S11 Expression of LAP by iTregs.** iTregs were stained with flow cytometric antibody of Foxp3/ LAP (iTreg) or Foxp3/isotype of LAP (isoType), then the expression of Foxp3 and LAP was analyzed by flow cytometry. Data are presented as the mean ±SD. Statistical significance was determined by Student's *t* test. ****P* < 0.001, ns=no significance. n=3.

**Figure S12**


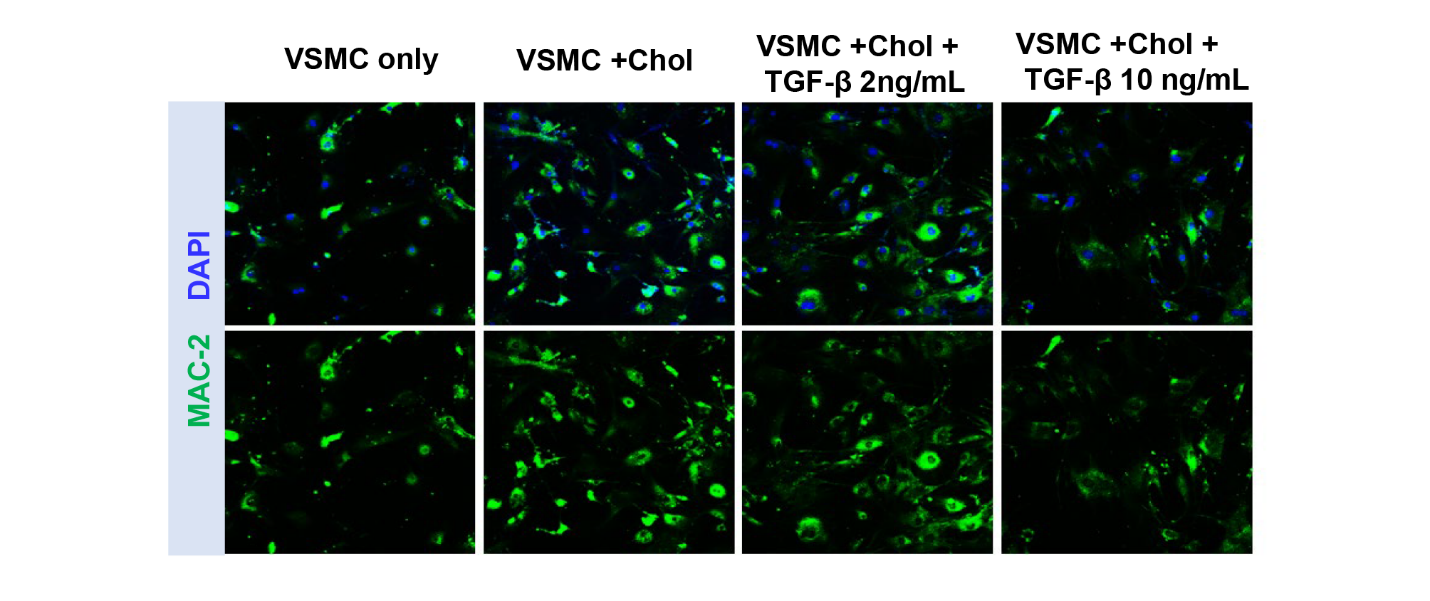


**Figure S12 TGF-β reduce macrophage-like trans-differentiation of VSMCs.** VSMCs were stimulated with cholesterol and treated with TGF-β for 48 h. VSMCs stimulated with cholesterol without TGF-β treatment for 48h is served as positive control (Control), VSMCs culture alone received no stimulation or treatment for 48h is served as a negative control (VSMC only). The expression of galectin-3 (Green) and DAPI (Blue) in VSMCs was detected by Immunofluorescence.

**Figure S13**


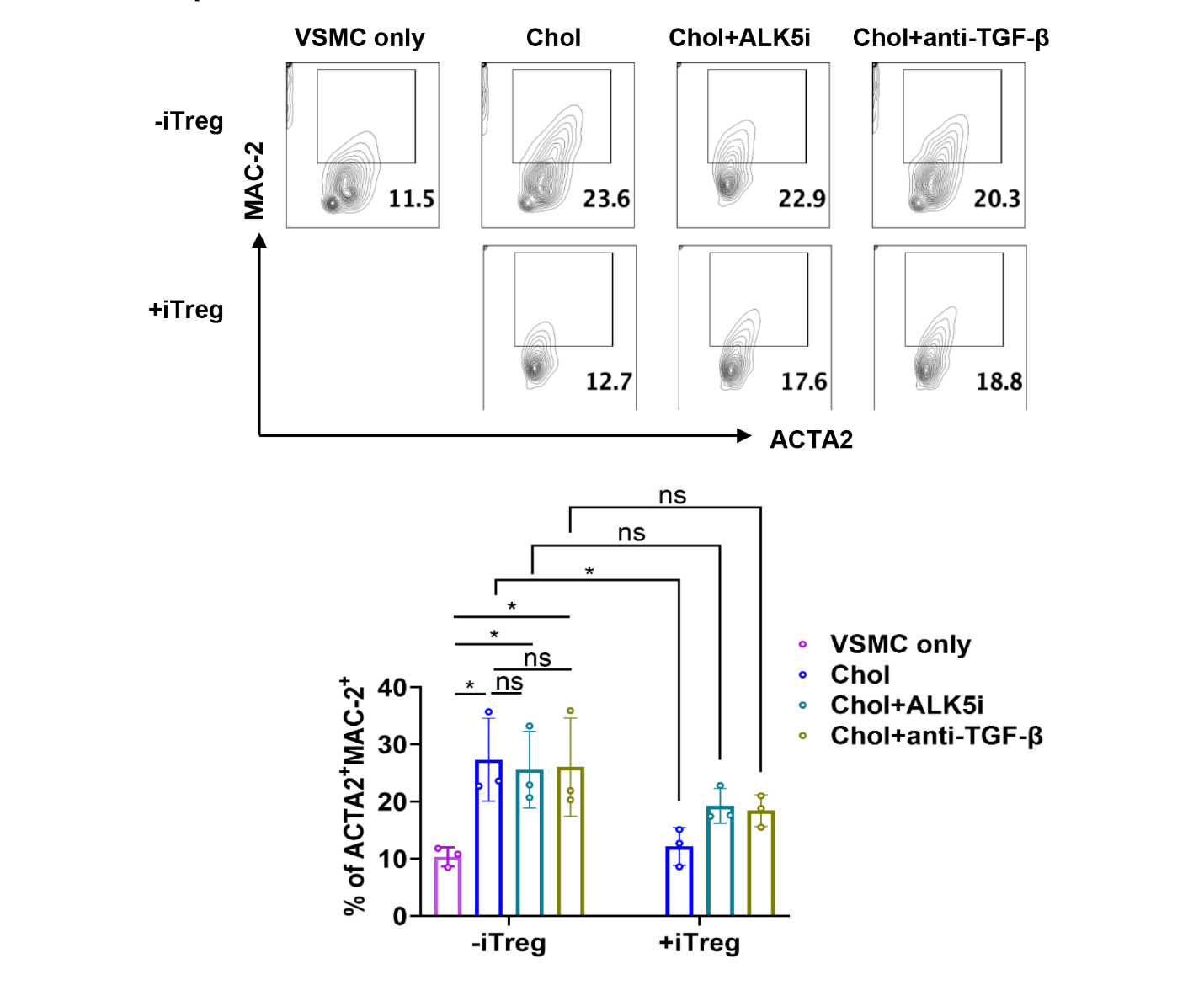


**Figure S13 Effect of iTreg Treatment on VSMCs Phenotype Switching partly via TGF-β Signaling**

VSMCs were co-cultured with iTregs or not, in the presence of cholesterol for 48 hours. In some experiments, TGF-β signaling was blocked by adding an activin-like kinase 5 inhibitor (ALK5i, LY3200882, 5 μM) or a TGF-β neutralizing antibody (anti- TGF-β, MA5-23795, 1 μg/ml). VSMCs from different experimental groups were collected, and the protein expression levels of ACTA2 and MAC-2 were analyzed by flow cytometry. Data are presented as the mean ± SD. Statistical significance was determined by Two-way ANOVA followed by multiple comparison tests. **P*< 0.05, ns=no significance. n=3.

**Figure S14**


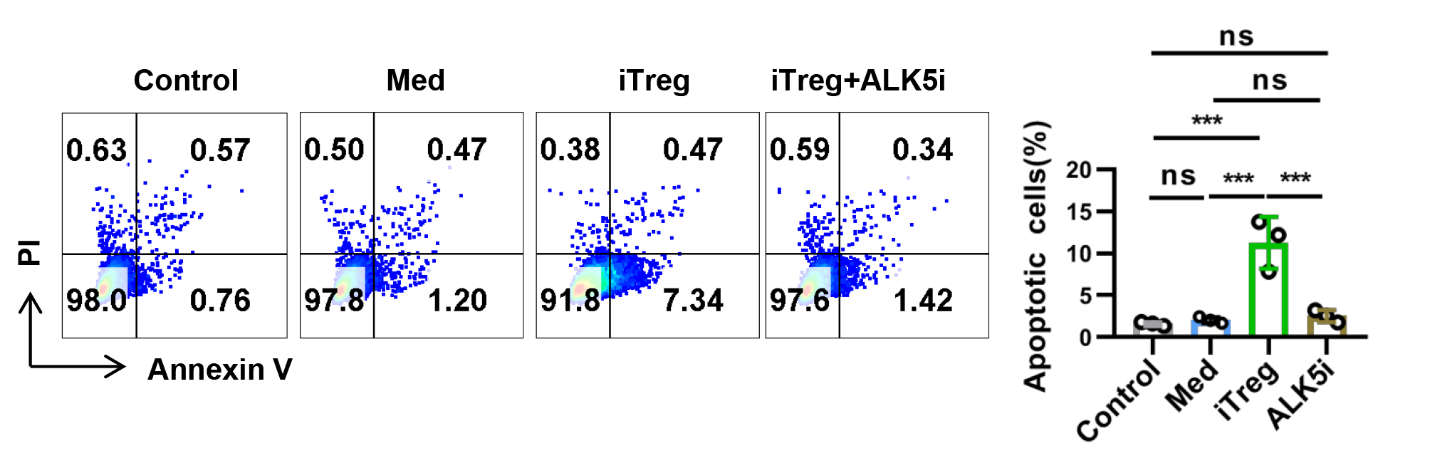


**Figure S14 iTregs facilitate VSMCs apoptosis mediated by TGF-β signaling.**

VSMCs were cultured in isolation (Control) or co-cultured with either control cells (Med) or iTregs (iTreg) in the presence of cholesterol for 48 hours. In certain experiments involving iTreg co-culture, TGF-β signaling was inhibited by the addition of an activin-like kinase 5 inhibitor (ALK5i, LY3200882, 5 µM). VSMCs from each treatment group were harvested and subjected to Annexin V/PI staining, followed by flow cytometric analysis. Data are presented as mean ± SD. Statistical significance was determined by One-way ANOVA followed by Sidak’s multiple comparisons. ****P* < 0.001, ns=no significance. n=3.

**Table S1 Murine specific primers**

| Gene | Forward primers (5' - 3') | Reverse primers (5' - 3') |
| --- | --- | --- |
| *Acta* | GTCCCAGACATCAGGGAGTAA | TCGGATACTTCAGCGTCAGGA |
| *Myh11* | GGAATCCTTTGGAAATGCGAAGA | GCCCCAACAATATAGCCAGTTAC |
| *Calponin* | *TCTGCACATTTTAACCGAGGTC* | *GCCAGCTTGTTCTTTACTTCAGC* |
| *Tropoelastin* | *TTGCTGATCCTCTTGCTCAAC* | *GCCCCTGGATAATAGACTCCAC* |
| *Lgals2* | *AGGAGAGGGAATGATGTTGCC* | *GGTTTGCCACTCTCAAAGGG* |
| *Lgals3* | *AACACGAAGCAGGACAATAACTGG* | *GCAGTAGGTGAGCATCGTTGAC* |
| *Cd68* | *CTTCCCACAGGCAGCACAG* | *ATGATGAGAGGCAGCAAGAGG* |
| *Il6* | *CAACGATGATGCACTTGCAGA* | *TGTGACTCCAGCTTATCTCTTGG* |
| *Mcp1* | *TCAGCCAGATGCAGTTAACGC* | *TCTGGACCCATTCCTTCTTGG* |
| *Il1b* | *TTCAGGCAGGCAGTATCACTC* | *CCACGGGAAAGACACAGGTAG* |
| *Mmp2* | CACACCAGGTGAACCATGTG | AGGGCTGCATTGCAAATATC |
| *Mmp3* | GTCCCTCTATGGAACTCCCAC | AGTCCTGAGAGATTTGCGCC |
| *Mmp12* | GCTGTCACAACAGTGGGAGA | ATGCTCCTGGGATAGTGTGG |
| *Mmp13* | TTGATGCCATTACCAGTCTCC | ACATGGTTGGGAAGTTCTGG |
| *Actb* | *GGCTGTATTCCCCTCCATCG* | *CCAGTTGGTAACAATGCCATGT* |
| *Gapdh* | *CCCTTAAGAGGGATGCTGCC* | *TACGGCCAAATCCGTTCACA* |
